# Cinnamaldehyde targets SarA to enhance β‐lactam antibiotic activity against methicillin‐resistant *Staphylococcus aureus*

**DOI:** 10.1002/mlf2.12121

**Published:** 2024-06-14

**Authors:** Jianguo Li, Tingyin Lu, Yuefei Chu, Yuejun Zhang, Jing Zhang, Wenzhen Fu, Jian Sun, Yahong Liu, Xiao‐Ping Liao, Yu‐Feng Zhou

**Affiliations:** ^1^ State Key Laboratory for Animal Disease Control and Prevention South China Agricultural University Guangzhou China; ^2^ Guangdong Provincial Key Laboratory of Veterinary Pharmaceutics Development and Safety Evaluation South China Agricultural University Guangzhou China; ^3^ Yantai Fushan Center for Animal Disease Control and Prevention Yantai China

**Keywords:** cinnamaldehyde, MRSA, SarA, β‐lactam resistance

## Abstract

Methicillin‐resistant *Staphylococcus aureus* (MRSA) is a current global public health problem due to its increasing resistance to the most recent antibiotic therapies. One critical approach is to develop ways to revitalize existing antibiotics. Here, we show that the phytogenic compound cinnamaldehyde (CIN) and β‐lactam antibiotic combinations can functionally synergize and resensitize clinical MRSA isolates to β‐lactam therapy and inhibit MRSA biofilm formation. Mechanistic studies indicated that the CIN potentiation effect on β‐lactams was primarily the result of inhibition of the *mecA* expression by targeting the staphylococcal accessory regulator *sarA*. CIN alone or in combination with β‐lactams decreased *sarA* gene expression and increased SarA protein phosphorylation that impaired SarA binding to the *mecA* promoter element and downregulated virulence genes such as those encoding biofilm, α‐hemolysin, and adhesin. Perturbation of SarA–*mecA* binding thus interfered with PBP2a biosynthesis and this decreased MRSA resistance to β‐lactams. Furthermore, CIN fully restored the anti‐MRSA activities of β‐lactam antibiotics in vivo in murine models of bacteremia and biofilm infections. Together, our results indicated that CIN acts as a β‐lactam adjuvant and can be applied as an alternative therapy to combat multidrug‐resistant MRSA infections.

## Impact statement

Methicillin‐resistant *Staphylococcus aureus* (MRSA) can frequently cause serious infections due to antibiotic resistance and biofilm formation that undermine clinical treatments. Therefore, alternative treatment strategies must be formulated due to the current lack of new classes of antibiotics. Adjuvant therapy that resensitizes bacterial pathogens to antibiotic action are a promising approach to this problem. We report that cinnamaldehyde (CIN) significantly potentiates the β‐lactam activity for MRSA infections, and even for biofilm eradication. Mechanistically, combination treatments with CIN and β‐lactams decreased *sarA*‐mediated virulence and *mecA*‐mediated resistance in MRSA by reducing the expression of the *sarA* gene and facilitating the phosphorylation of SarA protein. Perturbation of SarA–*mecA* binding by CIN interfered with PBP2a biosynthesis and contributed to the enhanced β‐lactam antibiotic activity against MRSA. These results indicate that CIN can be used as a potential treatment option to resensitize MRSA to the action of β‐lactam antibiotics.

## INTRODUCTION

Multidrug‐resistant (MDR) pathogens pose a serious global public health threat, with the rapid dissemination of methicillin‐resistant *Staphylococcus aureus* (MRSA). MRSA is highly toxigenic and can induce pore‐forming and exfoliative toxins that exacerbate skin and soft tissue infections, leading to bacteremia, endocarditis, and pneumonia^1^, ^2^. The therapeutic efficacy of most antibiotics against such bacterial infections is often compromised due to multiple resistance mechanisms^3^, ^4^, ^5^. For instance, the *mecA* gene encodes an alternative penicillin‐binding protein 2a (PBP2a) that shows low‐affinity to almost all β‐lactam antibiotics^6^. Previous studies have shown a positive correlation between the expression of *mecA* and the degree of β‐lactam resistance^7^. Conversely, inactivation of the *mecA* gene resensitized MRSA strains to β‐lactam antibiotics^8^. Interestingly, pharmacologic disruption of MRSA functional membrane microdomains (FMMs) using statin drugs can disable PBP2a oligomerization and restore β‐lactam susceptibility^9^. Thus, inhibiting *mecA* expression or PBP2a synthesis could be a promising strategy to combat MDR MRSA infections.

The staphylococcal *sarA* is a global regulator of MRSA virulence determinants and biofilm formation in vitro, and is also implicated in exacerbating disease severity in animal models of invasive infections^10^, ^11^, ^12^. Typically, *sarA*‐mediated MRSA biofilm formation contributes to significantly elevated resistance and tolerance to antibiotics^13^. Interestingly, previous studies demonstrated that *sarA* regulated β‐lactam antibiotic resistance in *mecA*‐positive MRSA^7^. For instance, growth rates of *sarA* mutant MRSA in the presence of oxacillin were much slower than their parental strains^14^. Thus, we speculated that pharmacologic disruption of *sarA* expression at either the pretranscriptional or posttranscriptional level would interfere with PBP2a synthesis and resensitize MRSA to β‐lactam antibiotics. In addition, the dual inhibition of both antibiotic resistance and virulence factors may prevent the resurgence of β‐lactam resistance and provide additional bactericidal activity against various MRSA phenotypes.

Cinnamaldehyde (CIN) is the major component, accounting for 70%, in the essential oils of *Cinnamomum cassia* Presl (*Lauraceae*)^15^. Previous studies have demonstrated potential antimicrobial and anti‐inflammatory activities of CIN^16^, ^17^, ^18^. For instance, addition of CIN to *Escherichia coli* cultures resulted in significant bacterial growth inhibition due to membrane disruption and oxidative damage^19^. In addition, CIN and substituted derivatives were previously reported to attenuate virulence in *Vibrio* spp. by targeting the LuxR quorum sensing response regulator and disrupting its DNA‐binding activity^20^.

In the current study, we examined whether CIN could restore β‐lactam antibiotic activities against MDR MRSA in vitro and in the murine models. Our experiments were successful in this respect, and we found that this potentiation effect was ascribed to the suppression of *sarA* expression and the increased phosphorylation level of SarA protein in the presence of CIN in combination with β‐lactams. This impaired both the abilities of SarA to bind the *mecA* promoter and to regulate virulence‐associated target genes, thus decreasing *mecA*‐mediated MRSA resistance to β‐lactam antibiotics. Our results indicate that CIN as a potential adjuvant may reestablish clinical use of older β‐lactams against MRSA and provide alternatives for the treatment of MDR MRSA infections.

## RESULTS

### CIN potentiates β‐lactam antibiotic activity against MRSA

Synthesis of PBP2a is a major mechanism accounting for MRSA resistance to β‐lactams^21^, ^22^, ^23^. In the current work, we chose the 18 clinical MRSA isolates that were resistant to ampicillin (AMP) and cefotaxime (CTX) with minimum inhibitory concentrations (MICs) of 4–256 and 16–512 mg/l, respectively (Tables S1 and S2). As expected, the isogenic *sarA* mutant strain (JE2 Δ*sarA*) showed a significant decrease in β‐lactam antibiotic MICs compared to the parental strain, consistent with increased susceptibility in the JE2 *mecA* mutant strain (JE2 Δ*mecA*) (Table S1). Interestingly, plasmid complementation of JE2 Δ*sarA* with either wild‐type *sar*A (pALC1215) or *mecA* (pALC6185) restored the MIC phenotypes to levels similar to those of the isogenic parent (Table S1).

**Figure 1:**
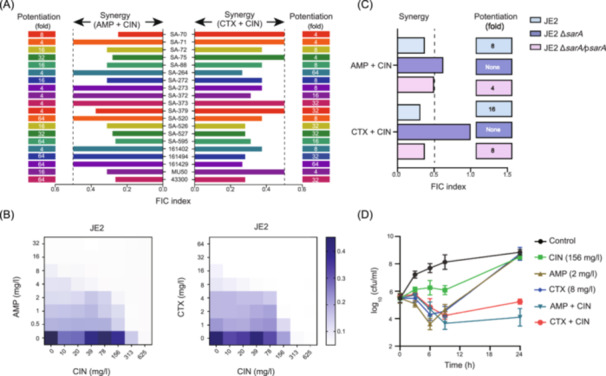
Cinnamaldehyde (CIN) potentiates the anti‐MRSA activity of β‐lactam antibiotics. (A) Synergic effects of CIN in combination with ampicillin (AMP) and cefotaxime (CTX) against clinical MRSA isolates derived from human bacteremia (SA‐372, SA‐373, SA‐520, SA‐595), wound exudates (SA‐70, SA‐71, SA‐72, SA‐75, SA‐88, SA‐264, SA‐272), and endotracheal aspirate (SA‐273, SA‐379, SA‐526, SA‐527, 161402, 161494, 161429). (B) Chequerboard microdilution assay results for CIN and AMP/CTX combinations against strain JE2. (C) FIC indices for combination treatments of JE2 parental, its isogenic JE2 Δ*sarA* mutant, and complemented JE2 Δ*sarA*/p*sarA* strains. Synergy is defined as an FIC index of ≤0.5. (D) Time–kill curves depicting in vitro potentiation of β‐lactams by CIN. Exponentially growing MRSA cells were challenged with sub‐inhibitory concentrations of CIN alone or in combination with AMP or CTX. Data represent values from three biological replicates. FIC, fractional inhibitory concentration; MRSA, anti‐methicillin‐resistant *Staphylococcus aureus*.

MRSA is typically associated with multiple infections and so we examined whether CIN can potentiate a β‐lactam antibiotic effect against a group of 18 clinical MRSA isolates derived from human bacteremia (4 isolates), wound exudates (7 isolates), and endotracheal aspirates (7 isolates). Similar synergistic effects were observed for CIN + AMP and CIN + CTX against all MRSA isolates and these combinations generated low fractional inhibitory concentration (FIC) indices of 0.25–0.5 (Figure 1A and Table S2). Interestingly, the FIC indices of CIN combined with AMP or CTX were <0.5 for the linezolid‐resistant and vancomycin‐intermediate strains (Table S2). In the presence of CIN, the activities of AMP and CTX were highly potentiated, as characterized by >4‐fold MIC reduction (Figure 1A,B). It is noteworthy that the JE2 Δ*sarA* mutant strain did not display synergism when CIN was used as a combination agent (FIC index ≥0.625). Thus, this synergistic potentiation by CIN was highly associated with *sarA*. As a further validation, the *sarA* complemented strain (JE2 Δ*sarA/*p*sarA*) restored the synergistic interaction of CIN and β‐lactams in the combinations (Figure 1C).

The increased activities of CIN in combination with AMP and CTX were further validated using the direct time–kill kinetic assays. Control cultures of MRSA JE2 as well as SA‐70 (wound exudate) and SA‐372 (bacteremia) increased by >3.0 log_10_ cfu/ml after 24 h of incubation. Despite a transient growth suppression, sub‐inhibitory levels of CIN, AMP, and CTX alone showed similar bacterial regrowth as the control group. In contrast, the presence of CIN + AMP and CIN + CTX combinations resulted in synergistic bactericidal effects of >3.0 log_10_ (cfu/ml) reduction compared to each drug alone against all MRSA strains (Figures 1D and S1). These results suggest that CIN may be a potent combination partner for boosting the activity of β‐lactam antibiotics against MRSA infections.

### CIN and β‐lactam antibiotic combinations inhibit *sarA*‐mediated MRSA biofilm formation

**Figure 2:**
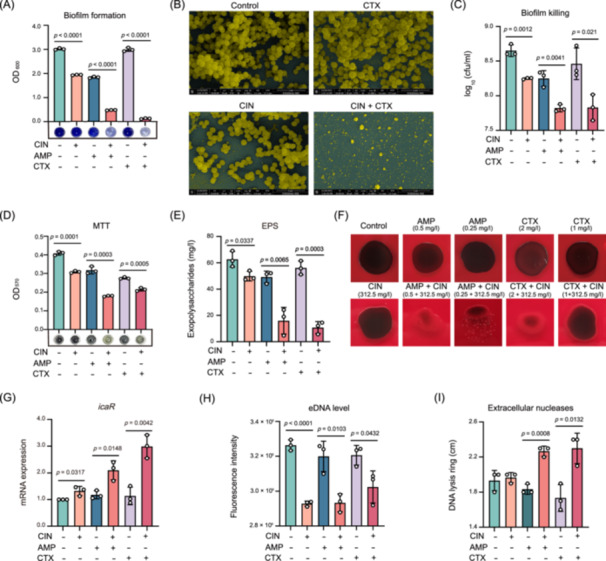
CIN and β‐lactam antibiotic combinations disrupt MRSA biofilm formation, viability, and matrix composition. MRSA JE2 strain tests using CIN and β‐lactams (AMP or CTX) alone or in combination. (A) Biofilm formation in the presence of CIN (312.50 mg/l), AMP (16 mg/l), CTX (32 mg/l) alone, or in combination. (B) Scanning electron microscopy (SEM) photomicrographs of biofilms formed in the presence of CIN (312.50 mg/l) and CTX (32 mg/l). (C, D) Measurements of biofilm‐embedded MRSA cells after single and combination therapies with CIN (156.25 mg/l), AMP (8 mg/l), and CTX (16 mg/l) using the colony‐forming unit (cfu) method (C) and the 3‐(4,5‐dimethylthiazol‐2‐yl)‐2,5‐diphenyltetrazolium bromide (MTT) metabolic assay (D). (E) Exopolysaccharide (EPS) production in an MRSA biofilm using single and combination therapies of AMP (4 mg/l), CTX (8 mg/l), and CIN (156.25 mg/l). (F, G) Polysaccharide intercellular adhesin (PIA) production (F) and PIA‐related *icaR* gene expression (G) for the indicated groups. (H) Extracellular DNA (eDNA) level of the indicated groups exposure to CIN (156.25 mg/l), AMP (8 mg/l), and CTX (16 mg/l) alone or in combination. (I) Extracellular nuclease activities of MRSA exposure to CIN(78.125 mg/l), AMP (4 mg/l), and CTX (8 mg/l), alone or in combination. Nuclease production was tested using DNase agar, and diameters of DNA lysis ring (cm) are indicated. Data represent the mean ± SD of three biological replicates. Statistical significances were determined using an unpaired Student's *t* test.

SarA is a key regulator of MRSA biofilm formation both in vitro and in vivo^24^. We therefore investigated whether CIN potentiation is conserved beyond planktonic cells and examined whether these combinations would alter MRSA biofilm formation capacity and composition. Interestingly, when combined with AMP or CTX, CIN resulted in significant decrease in JE2 biofilm formation compared to each drug alone (*p* < 0.0001; Figure 2A). These results were further confirmed by looser and thinner biofilm structures for the combination treatments in scanning electron microscopy (SEM) photomicrographs (Figure 2B). Combination treatments also even destroyed mature MRSA biofilms and resulted in similar significant reduction in viable bacterial count and metabolic activity detected by 3‐(4,5‐dimethylthiazol‐2‐yl)‐2,5‐diphenyltetrazolium bromide (MTT) assay compared to each monotherapy (Figure 2C,D).

MRSA biofilms consist of exopolysaccharide (EPS), polysaccharide intercellular adhesin (PIA), proteins, and extracellular DNA (eDNA) in variable proportions. Thus, we examined whether any of these individual biofilm components were preferentially targeted with CIN addition. The production of EPS in the combination groups ranged from 4.72 to 24.2 mg/l, with lower average EPS (13.39 mg/l) compared to the single‐drug groups (51.77 mg/l, *p* < 0.005; Figure 2E). Similar results were found for PIA production between single and combination groups (Figure 2F). In addition, the CIN and β‐lactam combinations significantly activated the expression of the *icaR* gene, a negative regulator of the *icaADBC* operon that represses PIA‐dependent biofilm production (*p* < 0.05; Figure 2G). In the presence of CIN, the amounts of eDNA were also markedly reduced compared to each drug alone and their respective controls (*p* < 0.05; Figure 2H). This is further confirmed by the higher level of extracellular nuclease production in DNase agar plates (Figure 2I). Overall, the combination of CIN with either AMP or CTX effectively inhibited MRSA biofilm formation by suppressing the biosynthesis of extracellular matrix components, including EPS, PIA, and eDNA.

### CIN and β‐lactam antibiotic combinations suppress MRSA virulence and inhibit infection progression

**Figure 3:**
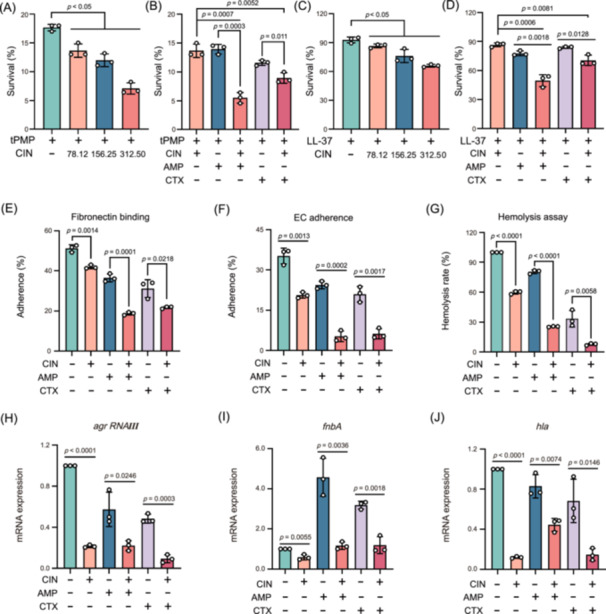
Impact of CIN and β‐lactam antibiotic combination on MRSA virulence and pathogenicity. (A–D) In vitro killing of MRSA JE2 by the host defense peptides thrombin‐induced platelet microbicidal protein (tPMP) (A, B) and cathelicidin LL‐37 (C, D) in the presence of CIN alone or in combination with AMP (8 mg/l) or CTX (16 mg/l). CIN level for combination treatments with AMP or CTX in panels (B) and (D) was 78.12 mg/l. (E, F) Adherence of MRSA to immobilized fibronectin (E) and EC (F) in the presence of CIN, AMP, CTX alone, or their combination. (G) Effects of CIN alone or in combination with AMP (0.25 mg/l) or CTX (0.5 mg/l) on hemolysin production of MRSA JE2. (H–J) Relative gene expression levels of *agr RNAIII* (H), *fnbA* (I), and *hla* (J) in the presence of sub‐MIC concentrations of CIN, AMP, CTX alone, or in combination. Data represent the mean ± SD of three biological replicates, and statistical significances were determined using an unpaired Student's *t* test. EC, endothelial cells.

An essential aspect of MRSA pathogenicity and necessary for the progression of bloodstream and skin infections involves evasion of killing by the innate host defense peptides (HDPs), such as thrombin‐induced platelet microbicidal protein (tPMP) and cathelicidin LL‐37^25^. We thus exposed MRSA cells to sub‐MIC CIN in the presence of tPMP or LL‐37, and found a clear concentration‐dependent decrease in bacterial survival for strain JE2 (Figure 3A,C). In particular, the combination of CIN and β‐lactams led to increased killing by tPMP or LL‐37 compared to the single‐drug groups (Figure 3B,D). Invasive MRSA must attach host cells and extracellular matrix ligands such as fibronectin to facilitate bacterial adhesion and internalization^26^. These abilities are crucial for establishing MRSA bacteremia. Interestingly, the presence of CIN + AMP and CIN + CTX combinations resulted in dramatic and significant (*p* < 0.05) decrease in MRSA binding to fibronectin (18.8%–21.8% vs. 31.2%–36.5%) and endothelial cells (5.33%–6.23% vs. 20.9%–24.4%) compared to single antibiotic treatments (Figure 3E,F). Moreover, the combined treatments showed synergistic inhibition of MRSA hemolysin activity (Figure 3G).

Consistent with these *sarA*‐related virulence phenotypes, the relative steady‐state mRNA expression of *agr RNAIII*, a key downstream regulator of *sarA*, and other virulence‐associated genes (*fnbA* and *hla*) were significantly inhibited after exposure to CIN alone or in combination with β‐lactams (Figure 3H–J). In particular, exposure to sub‐MIC levels of AMP or CTX resulted in increased *fnbA* expression compared to controls. In contrast, this effect was completely abolished when administered together with CIN (Figure 3I). These results suggest that CIN most likely alters the regulatory functions of *sarA*, consequently resulting in the inhibition of MRSA infection progression.

**Figure 4:**
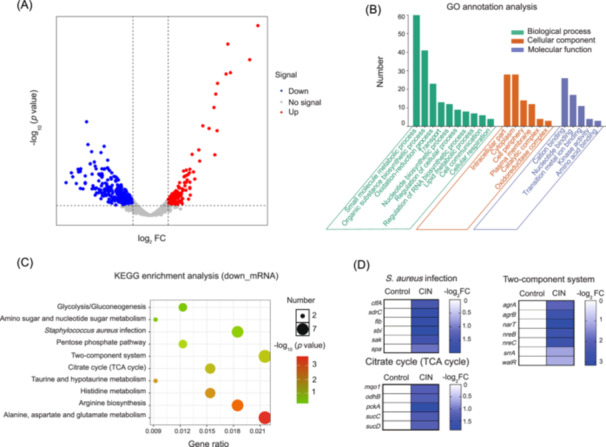
Transcriptome analysis of MRSA JE2 after exposure to sub‐MIC of CIN. (A) Volcano plot of the differentially expressed genes (DEGs) in MRSA JE2 treated with 156.25 mg/l CIN for 4 h. The *x* and *y* axes represent the FCs of expression and corresponding statistically significant degree, respectively. (B) Gene Ontology (GO) annotation classification analysis of downregulated DEGs in the biological process, cellular component, and molecular functions. (C) Kyoto Encyclopedia of Genes and Genomes (KEGG) enrichment analysis showing the 10 most significant biological pathways enriched in the downregulated DEGs. (D) Differential expression of selected downregulated genes associated with *S. aureus* infection, the citrate cycle, and a two‐component system. Data are presented as the means of three biological replicates. FC, fold change.

In order to obtain more insights into the molecular mechanism underlying CIN activity and CIN‐induced mRNA expression level changes, we performed a transcriptome analysis of strain JE2 following CIN exposure. We identified 446 differentially expressed genes (DEGs) between the CIN treatment and control groups, including 156 upregulated and 290 downregulated genes (Figure 4A and Table S3). Gene Ontology (GO) analysis of these DEGs provided major functional annotations for biological processes (small‐molecule metabolic and organic substance biosynthetic processes), cellular components (intracellular part), and molecular functions (cation and nucleotide binding) (Figure 4B). Kyoto Encyclopedia of Genes and Genomes (KEGG) enrichment analysis revealed that the markedly downregulated genes were associated with *S. aureus* infection and two‐component system pathways (Figure 4C). In particular, the genes encoding microbial surface components recognizing adhesive matrix molecules (MSCRAMM), such as *clfA* (clumping factor A), *spa* (staphylococcal protein A), *fib*, and *sdrC* (serine‐aspartate repeat protein C), were dramatically downregulated (Figure 4D). These reductions were associated with decreased MRSA adhesion to endothelial cells in the presence of sub‐MIC levels of CIN. Consistently, the expression of the staphylokinase (*sak*) and IgG‐binding protein (*sbi*) genes was reduced in the CIN treatment groups compared to the controls (Figures 4D and S2). We further verified the transcriptome results using quantitative real‐time‐PCR (qRT‐PCR), and their expression profiles were highly similar (Data not shown). These results suggest that CIN suppresses the secretion of virulence‐associated enzymes in MRSA and prevents production of components that compromise the host immune defense.

### CIN exposure alters SarA phosphorylation and binding to the *mecA* promoter that contributes to potentiation of anti‐MRSA activity for β‐lactam antibiotics

**Figure 5:**
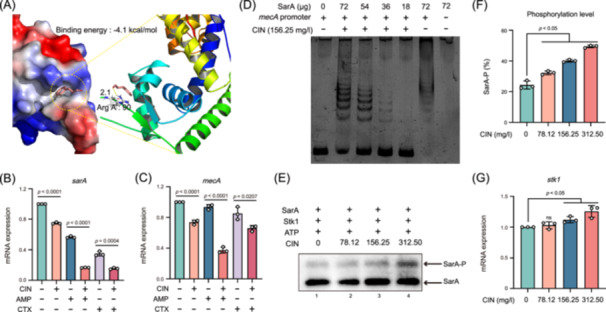
Synergistic mechanisms of CIN with β‐lactam antibiotics against MRSA. (A) Molecular docking analysis of interaction patterns of CIN to SarA protein. CIN interacts with Arg A: 90 (R90) in the DNA‐binding active site of SarA. (B, C) Relative expression levels of *sarA* (B) and *mecA* (C) in MRSA JE2 after exposure to CIN (156.25 mg/l) alone or in combination with AMP (8 mg/l) or CTX (16 mg/l). (D) Effects of CIN exposure on SarA‐*mecA* promoter binding. (E, F) SarA phosphorylation levels in the presence of CIN (0–312.50 mg/l). SarA: 2 μg; Stk: 1 µg; ATP: 1 mM. (G) Relative expression levels of *stk1* after exposure to CIN (0–312.50 mg/l). Data represent the mean ± SD of three biological replicates, and statistical significances were determined using an unpaired Student's *t* test.

Given that *sarA* is an important positive regulator of virulence factor expression, we thus speculated that the regulation of virulence genes by CIN is achieved through targeting SarA. Indeed, there is precedent for these types of CIN effects as has been demonstrated for LuxR^20^. As expected, CIN was able to decrease the expression of *sarA* in a concentration‐dependent manner (Figure S3) as well as directly interact with the SarA protein (Figure 5A). In particular, the arginine Arg A: 90 (R90) basic residue is a key target DNA site for SarA protein family members^27^. The in silico molecular docking analysis indicated that CIN could interact with the DNA‐binding active sites of SarA protein with a binding energy of −4.1 kcal/mol, and form a single Hydrogen‐bonding interaction with the R90 residue (Figure 5A). This interaction may alter the shape of SarA binding pockets when they bind to CIN. Addition of CIN + AMP and CIN + CTX combinations to MRSA cell cultures resulted in significant reduction of both *sarA* and *mecA* expression compared to either drug alone (*p* < 0.0005; Figure 5B,C). Interestingly, the *mecA* suppression was accompanied by *sarA* suppression. This suggests that *sarA* may positively regulate *mecA* expression by direct interaction of SarA protein at the *mecA* promoter. We examined this possibility via electrophoresis mobility gel shift assays (EMSAs) using *mec*A promoter element oligonucleotides and purified SarA protein. The presence of the SarA protein generated clear band‐shifts, indicating direct binding of SarA to the *mecA* promoter fragment. In particular, sub‐MIC levels of CIN remarkably reduced the binding of the *mecA* promoter to various concentrations of SarA protein (Figure 5D).

As a global regulator, SarA played an important role in target gene regulation at the posttranscriptional level. We therefore detected the phosphorylation level of SarA treated with CIN. In line with a previous study^28^, SarA was mainly phosphorylated by serine/threonine kinase Stk1 due to the inability to autophosphorylate in vitro (Figure S4A). As shown in Figure 5E,F, treatments with CIN increased the phosphorylation levels of SarA in the presence of Stk1 in a concentration‐dependent manner. A slight upregulation in *stk1* expression was observed after treatment with CIN (Figure 5G). Compared to monotherapy, the combinations of CIN and AMP/CTX demonstrated higher phosphorylation levels of SarA (*p* < 0.05; Figure S4B,C). It has been reported that phosphorylation of SarA weakens its DNA‐binding capacity^28^, ^29^. Thus, the effects of CIN alone or in combination with AMP/CTX, as demonstrated, are most likely due to a promotion of SarA phosphorylation that influences the differential binding of SarA to the *mecA* promoter, subsequently interfering with *mecA* expression. Together, these results indicated that treatments with CIN alone or in combination with β‐lactam antibiotics decreased *sarA*‐mediated virulence and *mecA*‐mediated resistance in MRSA by suppressing *sarA* expression and facilitating SarA phosphorylation.

### CIN enhances the efficacy of β‐lactam antibiotics in the murine models of MRSA bacteremia and subcutaneous catheter‐related biofilm infection

Therapeutic applications of antibiotics and plant‐natural compound combinations have been proven to be beneficial, as outlined in numerous clinical studies^30^, ^31^. However, novel combination therapies must first be evaluated for toxicity. To assess the potential treatment toxicity of CIN used as an antibiotic adjuvant, we analyzed hemolysis, histopathologic, and cytotoxicity of CIN alone or in combination with β‐lactams. The addition of CIN to blood cells at a high concentration of 12,500 mg/l did not result in any significant hemolytic activity, either alone or in combination with β‐lactams. Similarly, no visible cytotoxicity was observed in either HEK‐293 or Vero cells for these treatments (Figure S5). Furthermore, histologic evaluation of kidney tissues retrieved from the monotherapy and combination therapy groups revealed no significant nephrotoxicity compared to the controls (Figure S6). These data indicated that neither CIN nor the combination therapies showed toxicity, suggesting a good safety profile of CIN as a potential synergist to enhance β‐lactam antibiotic efficacy.

**Figure 6:**
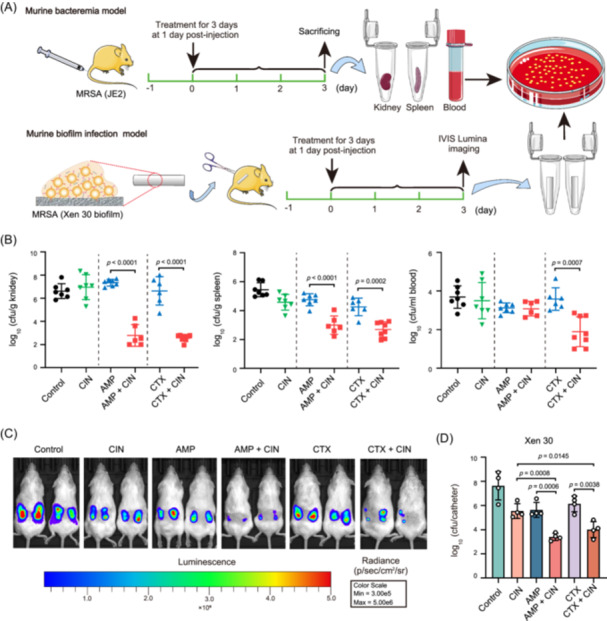
CIN enhances the efficacy of β‐lactam antibiotics in two murine infection models. (A) Experimental protocols for the murine models of bacteremia and subcutaneous catheter‐related biofilm infection due to MRSA. (B) Densities of MRSA JE2 strain in the kidney, spleen, and blood after a 3‐day β‐lactam antibiotic monotherapy or in combination with CIN in the murine bacteremia model. CIN at 20 mg/kg, intravenously (i.v.), once daily (qd); AMP at 40 mg/kg, orally (p.o.), three times daily (tid); CTX at 100 mg/kg, subcutaneously (s.c.), twice daily (bid). Each dot represents one mouse and the horizontal lines indicate the means of results from groups of six to eight mice. (C) Monitoring of therapeutic efficacy of AMP (80 mg/kg, p.o., tid), CTX (100 mg/kg, s.c., bid), or CIN (20 mg/kg, i.v., qd) monotherapy or combination therapy in a murine subcutaneous catheter‐related MRSA biofilm model using in vivo bioluminescent imaging. (D) Viable bacterial counts of MRSA attached on the catheters after monotherapy or combination therapy. Statistical significances were determined using an unpaired Student's *t* test. IVIS, in vivo bioluminescence imaging system.

MRSA is among the leading causes of biofilm colonization and bloodstream infections such as bacteremia and sepsis. Given the promising potentiation effect by CIN in vitro, we therefore investigated the potential of CIN to restore β‐lactam antibiotic efficacy in the murine bacteremia and catheter‐related biofilm infection models (Figure 6A). In the bacteremia model, mice infected with strains JE2 and MW2 did not respond to CIN or the β‐lactams AMP and CTX monotherapies, and MRSA densities in target tissues were similar to those in the respective untreated controls (Figures 6B and S7). Importantly, the CIN + AMP and CIN + CTX combination treatments resulted in highly significant (*p* < 0.0001) reduction of MRSA densities in kidneys (>4.1 log_10_ [cfu/g]) and spleens ( > 1.7 log_10_ [cfu/g]) of infected mice compared to each monotherapy (Figure 6B). In the murine catheter‐related biofilm infection model, both bioluminescence intensity and MRSA burden in catheters in the untreated controls increased considerably at 3 days after biofilm colonization (Figure 6C). At the end of therapy, despite the lower MRSA density, CIN, AMP, or CTX monotherapy did not reduce the MRSA biofilm burden compared to the initial colonization (~10^6^ cfu/catheter; Figure 6D). Interestingly, the CIN + AMP and CIN + CTX combination therapies resulted in >2.2 log_10_ cfu reduction of MRSA in catheters compared to the monotherapy groups. Similarly, the bioluminescence from mice receiving the combination treatments with CIN + AMP and CIN + CTX showed a significant decrease compared to that from each monotherapy group (Figure 6C,D).

## DISCUSSION

Clinical treatment options for MDR MRSA infections are limited to last‐line antibiotics such as vancomycin and daptomycin. This problem has been exacerbated by the recent emergences of vancomycin‐intermediate and ‐resistant *S. aureus* (VISA and VRSA)^32^. Thus, effective therapeutic strategies are urgently needed against such resistant pathogens. A feasible approach to overcome bacterial resistance is to develop bioactive adjuvants to revitalize existing antibiotics. A well‐known paradigm of this strategy is the coadministration of β‐lactam antibiotics (e.g., amoxicillin) alongside β‐lactamase inhibitors (e.g., clavulanic acid) that helps to neutralize β‐lactamases^33^. Other studies also reported diverse adjuvants targeting bacterial MDR mechanisms to increase the efficacies of existing antibiotics^30^, ^34^. For example, the active compounds pterostilbene and metformin could effectively restore the activities of carbapenem and tetracycline against MDR bacteria, respectively^35^, ^36^. These approaches were similar to our current work demonstrating a potentiation effect from CIN and β‐lactam antibiotic combinations.

**Figure 7:**
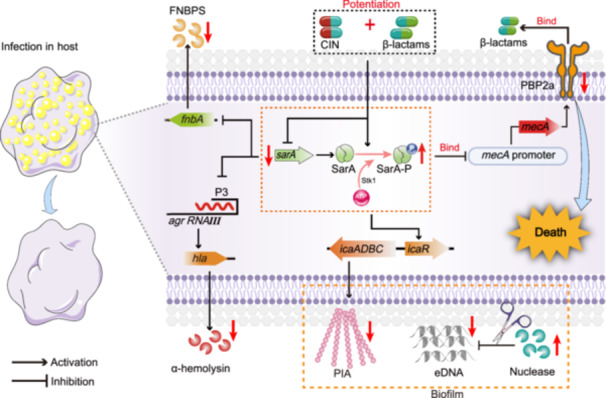
Mechanistic insight into the synergistic interaction of CIN in combination with β‐lactams against MRSA. FNBPS, fibrinogen binding proteins; PIA, polysaccharide intercellular adhesin.

The importance of global regulatory locus *sarA* in β‐lactam antibiotic resistance has been reported previously in vitro and in an experimental endocarditis infection model^7^. The present study further investigated whether the synergistic effect of CIN and β‐lactams was associated with the *sarA* regulon. We demonstrated that the *sarA* mutation rendered MRSA JE2 more susceptible to AMP and CTX killing compared to the parental strain. Complementation of the *sarA* mutant strain with *mecA* restored β‐lactam resistance. We also found significant synergy for this combination treatment against all clinical MRSA strains, except for the *sarA* knockout mutant. These results suggest that *sarA* is a promising target for the development of antibiotic adjuvants against MDR MRSA infections. In addition, the present study showed that CIN could inhibit *sarA* expression in a concentration‐dependent manner in the range of 78.12–312.50 mg/l (Figure S3). In particular, the level of *sarA* inhibition by the high concentration of CIN at 312.50 mg/l was comparable to that observed with 156.25 mg/l CIN when used in combination with AMP or CTX (Figures 5B and S3). We thus speculated that the disorganization of the cell wall by AMP or CTX may increase MRSA exposure to CIN^37^. This speculation was subsequently corroborated by our preliminary analysis using high‐performance liquid chromatography (HPLC), which demonstrated higher CIN accumulation in MRSA cells in the presence of AMP or CTX (data not shown). More importantly, a significant reduction in *sarA* expression was observed for all CIN‐based combination therapies compared to each monotherapy. These results indicated that *sarA* inhibition by CIN + AMP and CIN + CTX combinations may play an important role in triggering this potentiation effect. Besides offering mechanistic insights into transcription regulation, our studies revealed that CIN promoted the phosphorylation level of SarA and interfered with SarA binding to the *mecA* promoter, resulting in the inhibition of PBP2a expression encoded by *mecA* (Figure 7). This process thereby re‐sensitized MRSA to β‐lactam therapies.

MRSA is recognized as a common pathogen in both nosocomial and community settings, often infecting soft tissues and forming tenacious biofilms on these surfaces^38^. Biofilm formation enables further MRSA colonization of host tissues and provides an antibiotic‐impenetrable structure that also acts to exclude host immune factors^39^. Previous studies demonstrated that *sarA* was a positive regulator of MRSA biofilm formation because deletion of the *sarA* gene resulted in a loose biofilm architecture^7^, ^40^, ^41^. This phenotype was partly due to lack of the positive regulation of the *icaADBC* operon by *sarA* resulting in decreased levels of poly‐N‐acetyl‐β‐(1‐6)‐glucosamine that functions as the PIA^42^. Furthermore, biofilm‐associated protein (Bap) expression was also activated by *sarA* via an *agr*‐independent mechanism^43^. Consistently, our results indicated that CIN alone or in combination with β‐lactams effectively reduced the levels of EPS, eDNA, and PIA. In parallel, the combinations of CIN and β‐lactams reduced the steady‐state levels of *sarA* mRNA, resulting in increased expression of the *icaR* repressor of the *icaADBC* operon.

Numerous studies suggest that *sarA* is a global transcriptional regulator of virulence genes including *fnb*, *hla*, *cap5*, and *agr*, and plays a key role in MRSA infection establishment^43^, ^44^. In particular, MRSA capability to circumvent elimination by locally secreted HDP is a necessary step for bacteremia^45^. Furthermore, *sarA* is a regulator of adhesion and internalization in cases of invasive MRSA infections^46^, ^47^. Previous studies demonstrated that SarA protein positively modulated the production of fibrinogen binding protein (FnBP) by binding to the *fnbA* promoter fragments, thus facilitating adhesions to extracellular matrix ligands (fibronectin and fibrinogen) and to endothelial cells and platelet surface proteins^24^, ^47^. However, there is an inconsistent result that AMP or CTX induced *fnbA* expression but repressed *sarA* expression in this study (Figures 3I and 5B). This result may be attributed in part to the complexity of the *sarA* virulence regulon, and repression of *sarA* may have pleiotropic effects that are difficult to link to a specific virulence regulatory pathway for *fnbA*. Thus, *fnbA* may be regulated by multiple upstream factors in addition to *sarA*, and this is consistent with previous findings^48^, ^49^, ^50^. Importantly, CIN in combination with AMP or CTX decreased the expression of these pathogenic virulence genes including *fnbA* and *sarA* and resulted in consistent phenotypic observations. In addition, CIN‐treated MRSA cells showed higher susceptibility to the cationic HDPs (tPMP and LL‐37) and β‐lactam killing in vitro, reduced fibronectin adherence and minimal damage to endothelial cells, unstable biofilm, and decreased α‐hemolysin levels. These findings indicated that CIN at sub‐MIC concentrations clearly downregulated virulence factor expression that enhanced the efficacy of β‐lactam antibiotics against MRSA.

Our results also add to the growing body of research that helps explain improved clinical outcomes with the use of multifunctional natural products^51^. However, we did observe an unsatisfactory result from the AMP and CIN combination therapy in blood versus AMP monotherapy (Figure 6B). In addition to MRSA enrichments in the kidney and spleen, this diminished bacterial response to AMP‐based combination therapy in blood was most likely a result of the shorter half‐life and lower area under the concentration–time curve in plasma for AMP compared to CTX^52^, ^53^, ^54^. Nevertheless, all combination therapies achieved uniform significant reduction of MRSA densities in both the kidney and spleen compared to each monotherapy in the murine bacteremia model. Repurposing CIN from its current use as a food flavoring agent to a SarA inhibitor dramatically interfered with MRSA biofilm formation. This is especially important since biofilm‐associated infections are typically resistant to antibiotic therapy. As a result, CIN‐treated MRSA biofilms became susceptible to β‐lactam antibiotic therapies in a murine model of catheter‐related biofilm infection. Similarly, a previous study has also reported that Δ*sar*A MRSA strains are highly susceptible to vancomycin therapy in a rabbit endocarditis model^7^. In addition, our work contributes a context for the transformation of in vitro studies into in vivo treatment outcomes. CIN‐mediated suppression of MRSA virulence factors occurs predominantly by targeting SarA but is not bactericidal activity when used alone; rather, CIN functions by promoting SarA phosphorylation and perturbing SarA regulation of *mecA* and synergizes with β‐lactams to kill MRSA. The unique aspect of this approach is the targeting of a global regulator of MRSA and in general, virulence determinants that affect numerous infection‐related processes, and could thus be used in the development of alternative treatment strategies for MDR MRSA infections.

In summary, we demonstrated that β‐lactam antibiotic activity was increased by the presence of CIN against MRSA and biofilm‐associated infections both in vitro and in vivo. The combinations of CIN and β‐lactams inhibited *sarA* gene expression and increased SarA protein phosphorylation. This impaired SarA–*mecA* binding capacity and thus contributed to CIN potentiation effects on β‐lactam antibiotics. Together, our data suggest that CIN represents a promising β‐lactam antibiotic adjuvant to combat MDR MRSA infections and inspires us to find more effective candidates for use as potential antibiotic adjuvants.

## MATERIALS AND METHODS

### Chemicals and reagents

CIN (>95% purity) was purchased from Yuanye Biotechnology Company Ltd. and dissolved in dimethyl sulfoxide. AMP and CTX powders were purchased from Sigma.

### Bacterial strains and growth medium

MRSA strains used in this study are listed in Tables S1 and S2. Strain JE2 is a plasmid‐cured derivative from the community‐associated MRSA (CA‐MRSA) LAC (USA300) and its isogenic *mecA* mutant strains were obtained from the Nebraska Transposon Mutant Library (NTML)^7^, ^55^. A *sarA* gene knockout in JE2 (JE2 Δ*sarA*) was generated by transduction of the *sarA::kan* mutation from strain ALC2543 with phage 85^7^. Strain JE2 Δ*sarA* was complemented by transformation with plasmids pALC1215 (JE2 Δ*sarA/*p*sarA*) and pALC6185 (JE2 Δ*sarA*/p*mecA*) as described previously^7^, ^24^, ^56^. The JE2 and its isogenic mutant and complemented strains were used for subsequent mechanistic studies. CA‐MRSA strain MW2 (USA400) was used for in vivo experiments. An additional 18 clinical MRSA isolates for the checkerboard assays were derived from human bacteremia, wound exudates, and endotracheal aspirate (Table S2)**.** All strains were routinely grown, subcultured, and quantified using tryptic soy broth (TSB; Becton Dickinson) or on TSB agar plates. Brain–heart infusion broth (BHI) was used for biofilm assays.

### In vitro susceptibility testing and checkerboard test

The MICs of CIN, AMP, and CTX against MRSA strains were determined using the Clinical and Laboratory Standards Institute (CLSI) microdilution method^57^. All MIC determinations were performed in duplicate with three biological replicates. The median MIC of replicate assays was reported. Chequerboard tests were performed to assess interactions between CIN and β‐lactam antibiotics in standard 96‐well plates as reported previously^58^. An FIC index of ≤ 0.5 indicates a synergistic effect^59^.

### In vitro time–kill curves

Time–kill experiments were performed to evaluate the synergistic activity of CIN and β‐lactam antibiotic combinations as previously described^30^. Briefly, we used an initial inoculum of ~10^5.5 ^cfu/ml of logarithmic‐phase MRSA cells in the presence of sub‐MICs of CIN alone or in combination with AMP or CTX. Serial dilutions of MRSA cultures obtained at 0, 3, 6, 9, and 24 h were taken for bacterial density measurement using the plate count technique. Synergistic activity was considered as a reduction of ≥2 log_10_ (cfu/ml) at 24 h by the combination compared to each drug alone^58^.

### Biofilm formation, viability, and microscopic analysis

Exponential MRSA cells were adjusted to an OD_600_ of 0.5 and diluted 1:100 into BHI broth supplemented with 0.5% glucose^26^. The suspensions were transferred to six‐well plates containing CIN (312.50 mg/l) alone or in combination with AMP (16 mg/l) or CTX (32 mg/l) and incubated for 72 h at 37°C. After incubation, the plates were then gently washed with phosphate‐buffered saline (PBS), air‐dried, and stained using 0.1% crystal violet. The adhering dye was dissolved in 30% acetic acid and absorption was measured at OD_600_ to quantify biofilm formation. For the mature MRSA biofilm cultured for 72 h, the capacity of CIN (156.25 mg/l), AMP (8 mg/l), and CTX (16 mg/l) alone or in combination to kill biofilm‐embedded bacteria was determined by viable bacterial count and MTT metabolic assays^60^, ^61^. Biofilm formation was confirmed by SEM as previously described^18^, ^62^. In brief, pieces of plate material were fixed with 2.5% glutaraldehyde and 0.1% OsO_4_ for 1 h, chemically dried in a graded ethanol series (30%‐100%), and sputter‐coated with gold for SEM to analyze adherence and biofilm formation.

### EPS, eDNA, and nuclease production

MRSA biofilms were treated with CIN (156.25 mg/l), AMP (4 mg/l), and CTX (8 mg/l) alone or in combination. After treatment, biofilms were rinsed, removed, and dispersed by sonication at 20 kHz for 5 s. The suspension was collected for EPS determinations and quantified using the anthrone‐sulfuric acid colorimetric assay^26^, ^63^. Release of eDNA was determined from a 72 h biofilm treated with CIN (156.25 mg/l), AMP (8 mg/l), and CTX (16 mg/l) alone or in combination using a microplate fluorescence assay with Hoechst 33258 dye^26^, ^64^. Extraction and purification of biofilm eDNA were performed as previously described^64^. The eDNA was quantified using an EnSight fluorescence plate reader (Perkin Elmer) at Ex_350_/Em_460_. Extracellular nuclease activities of MRSA after exposure to CIN (78.12 mg/l), AMP (4 mg/l), and CTX (8 mg/l) alone or in combination, were determined using the DNase test agar^26^.

### PIA assay

Colony morphology on Congo red agar (CRA) was used as a surrogate marker of PIA production as described previously^65^. To assess the effect of CIN on slime production as characterized by PIA, a 10 µl aliquot of bacterial solution (OD_600_ = 1.2) was inoculated on CRA plates containing 0.25–0.5 mg/l AMP and 1–2 mg/l CTX alone or in combination with CIN (312.50 mg/l), and the plates were observed after incubation for 24 h at 37°C^66^.

### In vitro susceptibility to HDPs

Our previous study of in vitro HDP susceptibilities to MRSA and in vivo treatment outcomes involved the use of tPMP and cathelicidin LL‐37^26^. We therefore used these peptides in this study, and the tPMP preparation and bioactivity assays were performed as detailed elsewhere^67^. Cathelicidin LL‐37 was purchased from Eurogentec. MRSA cells were treated with/without CIN (78.12–312.50 mg/l), AMP (8 mg/l), and CTX (16 mg/l) alone or in combination for 2 h. After the pretreatment, cells were gently washed three times with PBS. In vitro HDP susceptibilities were assessed by adding tPMP (1 mg/l equivalent) to 10^4^ cfu/ml MRSA cells and LL‐37 (5 mg/l) to 10^5^ cfu/ml MRSA cells. The peptide concentrations were selected based on the pilot studies identifying their levels that did not rapidly kill MRSA cells over 2 h incubation period^26^. The results were expressed as the percentage (±SD) of the initial bacterial inoculum that survived under HDP exposure.

### Measurement of adherence to fibronectin and endothelial cells

To measure the effect of CIN on the ability of MRSA to adhere to fibronectin, six‐well plates were coated with 50 mg/l purified human fibronectin (Sigma) overnight at 4°C and washed with PBS. Plates were then treated with 3% bovine serum albumin to prevent nonspecific adhesion^25^, ^26^. Logarithmic‐phase MRSA cells were pretreated with/without CIN (156.25 mg/l), AMP (8 mg/l), and CTX (16 mg/l) alone or in combination for 2 h, and then added at a final inoculum of 5 × 10^3^ cfu/ml to fibronectin‐coated plates. Unbound MRSA cells were removed by gently washing three times with PBS, and melted TSA was added to each well and allowed to solidify at 37°C. The level of adherence was quantified by determining the percentage of the initial bacterial inoculum bound to fibronectin.

Human aortic endothelial cells (HAECs) were prepared and cultured as previously described^68^, ^69^. After pretreatment with CIN (39.06 mg/l) alone or in combination with AMP (0.25 mg/l) or CTX (0.5 mg/l), MRSA cells (5 × 10^5^ cfu/ml) were added to confluent endothelial cell monolayers in six‐well plates (MOI = 1:1) and incubated for 2 h under static conditions^26^. Cells were washed with Hanks balanced salt solution to remove the unbound bacteria, followed by permeabilization with 1.0% Triton X‐100, after which bacterial counts were directly determined by plating the appropriate serial dilutions on TSA. Adherence was calculated as the percentage of the initial bacterial inoculum bound to HAEC cells.

### Hemolysis quantification assay

The hemolytic activity of MRSA strains after exposure to CIN alone or in combination with β‐lactam antibiotics was determined as previously described^70^, ^71^. Briefly, culture supernatants lacking bacterial cells were collected from control and drug‐treated (39 mg/l of CIN, 0.25 mg/l of AMP, and 0.5 mg/l of CTX alone or in combination) MRSA strains by centrifugation at 6000 rpm for 10 min. Hemolytic activity was determined by incubating the cell‐free culture supernatants with an equal volume of defibrillated sheep blood cells (8% in PBS) for 20 min at 37°C. After incubation, the absorbance of released hemoglobin in the supernatant was determined at OD_543 _to obtain the hemolysis rate (HR) using the following equation^70^: HR(%) = OD_treated_/OD_control_ × 100%.

### Transcriptomic analysis

To determine the gene expression profile of MRSA in the presence of CIN, MRSA JE2 cultures were treated with or without CIN (156.25 mg/l) for 4 h. Total RNA was extracted using an EZNA bacterial RNA kit (Omega Biotek) and paired‐end sequenced with a read length of 2 × 150 bp on an Illumina NovaSeq. 6000 system (Novogene). Raw sequencing reads were filtered and mapped against the *S. aureus* NCTC 8325 reference genome (NC_007795.1). A Cuffdiff analysis was performed based on FPKM (Fragments per Kilobase of transcript per Million mapped reads) to identify DEGs between the control and the treatment group. Criteria for selecting DEGs were fold change (FC) values > 2 and *p* < 0.05. Three biological replications were conducted for each treatment.

### qRT‐PCR analysis

Exponential phases of MRSA cells were pretreated with CIN (78.12–312.50 mg/l) alone or in combination with AMP (8 mg/l) or CTX (16 mg/l). Total RNA was extracted from MRSA cells using the RNeasy extraction kit (Qiagen) as per the manufacturer's instructions. A HiScript III 1st Strand cDNA Synthesis Kit (Vazyme) was used for cDNA synthesis. Gene expression was quantified by qRT‐PCR using Taq Pro Universal SYBR qPCR Master Mix (Vazyme). Three biological replicates each containing three technical replicates were carried out for each gene. Amplification of *sarA*, *mecA*, *icaR*, *fnbA*, *RNAШ*, *hla*, and *stk1* genes was performed as previously described in detail, using gene‐specific primers to verify transcriptome results^30^. The relative abundance of *gyrB* mRNA was used as an internal control.

### Molecular docking analysis

To evaluate the binding interactions of CIN with the SarA protein, AutoDock v. 4.2.6 (https://autodock.scripps.edu/) was used for in silico docking analysis. The initial X‐ray crystal structure of the SarA protein was obtained from the Protein Data Bank (https://www.rcsb.org/) with PDB code 2FRH. The CIN chemical structure (C_9_H_8_O; PubChem ID: 637511) was obtained from the NCBI PubChem database (https://pubchem.ncbi.nlm.nih.gov/). The outputs from AutoDock and all modeling studies as well as images were rendered with PyMol v. 1.5.0.3 (https://pymol.org/2/).

### In vitro SarA phosphorylation assay

To determine the phosphorylation‐level changes of SarA treated with CIN alone (78.12–312.50 mg/l) or in combination with AMP (8 mg/l) or CTX (16 mg/l), the expression and purification of SarA and Stk1 proteins were performed as described previously in detail^72^. In brief, plasmid pET28a was transformed into *E. coli* BL21 (DE3) for the fusion protein expression, and His‐tagged SarA and Stk1 proteins were purified using an NGC chromatography system (BioRad). In vitro phosphorylation of purified SarA protein was performed as previously described, with some modifications^28^, ^73^. In brief, 2 μg of SarA was added to kinase buffer together with 1 mM ATP and 1 µg of Stk1 in the presence or absence of CIN (0–312.50 mg/l) for 20 min at 37°C. The reaction was terminated with addition of SDS loading buffer and heated at 95°C for 5 min. Subsequently, samples were separated on polyacrylamide gels supplemented with Phos‐Tag acrylamide. Protein mobility shift and intensity were quantified with ImageJ software (https://imagej.net/).

### The impact of CIN on SarA–*mecA* binding

EMSAs were used to investigate whether SarA directly regulates *mecA* expression and the effect of CIN on SarA–*mecA* promoter binding. Purified SarA protein and the 200 bp DNA fragment containing the *mecA* promoter region were incubated in the presence and absence of 156.25 mg/l CIN for 20 min at room temperature. The gel was stained with SYBR green using a commercially available EMSA kit (Invitrogen) and visualized by UV epi‐illumination at 302 nm.

### In vivo murine MRSA bacteremia model

Six‐week‐old specific‐pathogen‐free ICR mice (25–27 g) were obtained from Guangdong Medical Lab Animal Center (Guangzhou, China). The Animal Research Committee (IACUC) of the South China Agricultural University approved the animal studies (#2021C086). In the murine bacteremia model, mice were infected via the tail vein with a 0.25 ml MRSA bacterial suspension delivering ∼10^5.5^ cfu/mouse^26^. At 24 h postinfection, mice were randomized to receive (i) control treatment; (ii) CIN at 20 mg/kg intravenously (i.v.) once daily (qd); (iii) AMP at 40 mg/kg orally (p.o.) three times daily (tid); (iv) CTX at 100 mg/kg subcutaneously (s.c.) twice daily (bid); and (v) a combination of CIN with CTX or AMP. These doses were chosen to mimic pharmacokinetic values similar to those achieved by the clinical dosing of humans (i.e., 0.5 g AMP p.o. tid, and 1.0 g CTX i.v. bid)^53^, ^54^, ^74^, ^75^. Treatments lasted for 3 days using six to eight mice per group. After killing the mice, the target tissues (blood, kidney, and spleen) were removed, weighed, and quantitatively cultured for CFU determinations. MRSA burdens in target tissues were determined from each treatment, and expressed as the mean (±SD) of log_10_ (cfu/g or cfu/ml).

### In vivo murine model of subcutaneous catheter‐related MRSA biofilm infection

An in vivo bioluminescence imaging system (IVIS) was used for noninvasive monitoring of the therapeutic efficacy of CIN alone or in combination with AMP or CTX in a murine catheter‐related MRSA biofilm model^76^. Mice were infected by subcutaneously implanting a precolonized Teflon catheter segment (1 cm) inoculated with the *lux* bioluminescent MRSA strain Xen30 (Perkin Elmer) at ~10^6^ cfu/catheter^77^. At 1 day postimplantation, mice received either no therapy (control), CIN at 20 mg/kg i.v. qd, AMP at 80 mg/kg p.o. tid, and CTX at 100 mg/kg s.c. bid or their combinations. Mice were imaged after 3 days of implantation for bioluminescent signals (BLS) using the IVIS Lumina imaging system (PerkinElmer), which was presented as radiance (photons/s/cm^2^/steradian) using a pseudocolor scale (red, most intense; blue, least intense)^76^, ^78^. After killing the mice, the catheters were quantitatively cultured using the standard assays and reported as cfu/catheter^79^.

### Safety assessment

The effect of CIN alone or in combination with β‐lactam antibiotics on the hemolytic activity was determined as previously described^36^. In brief, a 2% sheep red blood cell suspension was incubated with CIN (1560–12,500 mg/l), AMP (1280 mg/l), and CTX (1280 mg/l) alone or in combination at 37°C for 1 h. Triton X‐100 (0.2%) and saline were used as positive and negative controls, respectively. The hemolytic activity (HA) was calculated according to the following equations: HA = 1−(OD_sample_−OD_negative_)/(OD _positive_−OD_negative_) × 100%. In vitro cytotoxicity of CIN alone or in combination with β‐lactam antibiotics against human embryonic kidney (HEK‐293) cells and Vero cells (2 × 10^5^ cells/well) was determined using the CCK‐8 assay^30^. For in vivo toxicity evaluation, mice receiving CIN (20 mg/kg) or CTX (100 mg/kg) monotherapy and combination therapy were sacrificed after 3 days of treatment, and their kidneys were harvested for hematoxylin and eosin staining using a standard staining protocol^80^. Mice treated with saline of the same volume served as controls.

### Statistical analyses

Statistical analysis was performed using GraphPad Prism 8.0 software. All data were presented as mean ± SD. Student's *t* test was used for statistical comparisons between groups. Differences with *p* ≤ 0.05 were considered statistically significant.

## AUTHOR CONTRIBUTIONS

**Jianguo Li**: Methodology (lead); writing—original draft (lead). **Tingyin Lu**: Methodology (supporting); visualization (equal). **Yuefei Chu**: Methodology (supporting); visualization (equal). **Yuejun Zhang**: Methodology (supporting). **Jing Zhang**: Methodology (supporting). **Wenzhen Fu**: Methodology (supporting). **Jian Sun**: Supervision (equal); visualization (equal). **Yahong Liu**: Funding acquisition (lead); supervision (equal). **Xiao‐Ping Liao**: Conceptualization (equal); supervision (lead). **Yu‐Feng Zhou**: Conceptualization (lead); funding acquisition (supporting); writing—review and editing (lead).

## ETHICS STATEMENT

Animal studies were approved by the Animal Research Committee (IACUC) of the South China Agricultural University (Guangzhou, China), approval number 2021C086.

## CONFLICT OF INTERESTS

The authors declare no conflict of interests.

## Supporting information

Supporting information.

Supporting information.

## Data Availability

The transcriptomic sequencing data have been deposited and are available at the NCBI SRA database under the BioProject reference PRJNA1008129.

## References

[1] Bassetti M , Baguneid M , Bouza E , Dryden M , Nathwani D , Wilcox M . European perspective and update on the management of complicated skin and soft tissue infections due to methicillin‐resistant *Staphylococcus aureus* after more than 10 years of experience with linezolid. Clin Microbiol Infect. 2014;20:3–18. 24580738 10.1111/1469-0691.12463

[2] Hassoun A , Linden PK , Friedman B . Incidence, prevalence, and management of MRSA bacteremia across patient populations‐a review of recent developments in MRSA management and treatment. Crit Care. 2017;21:211. 28807042 10.1186/s13054-017-1801-3 PMC5557425

[3] Boucher H , Miller LG , Razonable RR . Serious infections caused by methicillin‐resistant *Staphylococcus aureus* . Clin Infect Dis. 2010;51:S183–S197. 20731576 10.1086/653519

[4] Boucher HW , Sakoulas G . Perspectives on daptomycin resistance, with emphasis on resistance in *Staphylococcus aureus* . Clin Infect Dis. 2007;45:601–608. 17682996 10.1086/520655

[5] Boucher HW , Corey GR . Epidemiology of methicillin‐resistant *Staphylococcus aureus* . Clin Infect Dis. 2008;46:S344–S349. 18462089 10.1086/533590

[6] Peacock SJ , Paterson GK . Mechanisms of methicillin resistance in *Staphylococcus aureus* . Annu Rev Biochem. 2015;84:577–601. 26034890 10.1146/annurev-biochem-060614-034516

[7] Li L , Cheung A , Bayer AS , Chen L , Abdelhady W , Kreiswirth BN , et al. The global regulon *sarA* regulates β‐lactam antibiotic resistance in methicillin‐resistant *Staphylococcus aureus in vitro* and in endovascular infections. J Infect Dis. 2016;214:1421–1429. 27543672 10.1093/infdis/jiw386 PMC5079373

[8] Chen FJ , Wang CH , Chen CY , Hsu YC , Wang KT . Role of the *mecA* gene in oxacillin resistance in a *Staphylococcus aureus* clinical strain with a *pvl*‐positive ST59 genetic background. Antimicrob Agents Chemother. 2014;58:1047–1054. 24277044 10.1128/AAC.02045-13 PMC3910894

[9] García‐Fernández E , Koch G , Wagner RM , Fekete A , Stengel ST , Schneider J , et al. Membrane microdomain disassembly inhibits MRSA antibiotic resistance. Cell. 2017;171:1354–1367. 29103614 10.1016/j.cell.2017.10.012 PMC5720476

[10] Dunman PM , Murphy E , Haney S , Palacios D , Tucker‐Kellogg G , Wu S , et al. Transcription profiling‐based identification of *Staphylococcus aureus* genes regulated by the *agr* and/or *sarA* loci. J Bacteriol. 2001;183:7341–7353. 11717293 10.1128/JB.183.24.7341-7353.2001 PMC95583

[11] Chien Y , Manna AC , Cheung AL . SarA level is a determinant of *agr* activation in *Staphylococcus aureus* . Mol Microbiol. 1998;30:991–1001. 9988476 10.1046/j.1365-2958.1998.01126.x

[12] Cheung AL , Nishina KA , Trotonda MP , Tamber S . The SarA protein family of *Staphylococcus aureus* . Int J Biochem Cell Biol. 2008;40:355–361. 18083623 10.1016/j.biocel.2007.10.032 PMC2274939

[13] Yan J , Bassler BL . Surviving as a community: antibiotic tolerance and persistence in bacterial biofilms. Cell Host Microbe. 2019;26:15–21. 31295420 10.1016/j.chom.2019.06.002 PMC6629468

[14] Trotonda MP , Xiong YQ , Memmi G , Bayer AS , Cheung AL . Role of *mgrA* and *sarA* in methicillin‐resistant *Staphylococcus aureus* autolysis and resistance to cell wall‐active antibiotics. J Infect Dis. 2009;199:209–218. 19072553 10.1086/595740 PMC2782823

[15] Rao PV , Gan SH . Cinnamon: a multifaceted medicinal plant. Evid Based Complementary Altern Med. 2014;2014:1–12. 10.1155/2014/642942 PMC4003790 24817901

[16] Ahn SG , Jin YH , Yoon JH , Kim SA . The anticancer mechanism of 2′‐hydroxycinnamaldehyde in human head and neck cancer cells. Int J Oncol. 2015;47:1793–1800. 26352194 10.3892/ijo.2015.3152

[17] Chao LK , Hua KF , Hsu HY , Cheng SS , Lin IF , Chen CJ , et al. Cinnamaldehyde inhibits pro‐inflammatory cytokines secretion from monocytes/macrophages through suppression of intracellular signaling. Food Chem Toxicol. 2008;46:220–231. 17868967 10.1016/j.fct.2007.07.016

[18] Jia P , Xue YJ , Duan XJ , Shao SH . Effect of cinnamaldehyde on biofilm formation and *sarA* expression by methicillin‐resistant *Staphylococcus aureus* . Lett Appl Microbiol. 2011;53:409–416. 21767279 10.1111/j.1472-765X.2011.03122.x

[19] He TF , Wang LH , Niu D , Wen Q , Zeng XA . Cinnamaldehyde inhibit *Escherichia coli* associated with membrane disruption and oxidative damage. Arch Microbiol. 2019;201:451–458. 30293114 10.1007/s00203-018-1572-5

[20] Brackman G , Defoirdt T , Miyamoto C , Bossier P , Van Calenbergh S , Nelis H , et al. Cinnamaldehyde and cinnamaldehyde derivatives reduce virulence in *Vibrio* spp. by decreasing the DNA‐binding activity of the quorum sensing response regulator LuxR. BMC Microbiol. 2008;8:149. 18793453 10.1186/1471-2180-8-149 PMC2551610

[21] Fuda C , Suvorov M , Vakulenko SB , Mobashery S . The basis for resistance to β‐lactam antibiotics by penicillin‐binding protein 2a of methicillin‐resistant *Staphylococcus aureus* . J Biol Chem. 2004;279:40802–40806. 15226303 10.1074/jbc.M403589200

[22] Malouin F , Bryan LE . Modification of penicillin‐binding proteins as mechanisms of beta‐lactam resistance. Antimicrob Agents Chemother. 1986;30:1–5. 3530121 10.1128/aac.30.1.1 PMC176423

[23] Gonzales PR , Pesesky MW , Bouley R , Ballard A , Biddy BA , Suckow MA , et al. Synergistic, collaterally sensitive β‐lactam combinations suppress resistance in MRSA. Nat Chem Biol. 2015;11:855–861. 26368589 10.1038/nchembio.1911 PMC4618095

[24] Abdelhady W , Bayer AS , Seidl K , Moormeier DE , Bayles KW , Cheung A , et al. Impact of vancomycin on *sarA*‐mediated biofilm formation: role in persistent endovascular infections due to methicillin‐resistant *Staphylococcus aureus* . J Infect Dis. 2014;209:1231–1240. 24403556 10.1093/infdis/jiu007 PMC3969550

[25] Xiong YQ , Fowler, Jr. VG , Yeaman MR , Perdreau‐Remington F , Kreiswirth BN , Bayer AS . Phenotypic and genotypic characteristics of persistent methicillin‐resistant *Staphylococcus aureus* bacteremia in vitro and in an experimental endocarditis model. J Infect Dis. 2009;199:201–208. 19086913 10.1086/595738 PMC2827482

[26] Zhou YF , Li L , Tao MT , Sun J , Liao XP , Liu YH , et al. Linezolid and rifampicin combination to combat *cfr*‐positive multidrug‐resistant MRSA in murine models of bacteremia and skin and skin structure infection. Front Microbiol. 2020;10:3080. 31993042 10.3389/fmicb.2019.03080 PMC6971047

[27] Liu Y , Manna AC , Pan CH , Kriksunov IA , Thiel DJ , Cheung AL , et al. Structural and function analyses of the global regulatory protein SarA from *Staphylococcus aureus* . Proc Natl Acad Sci USA. 2006;103:2392–2397. 16455801 10.1073/pnas.0510439103 PMC1413715

[28] Didier JP , Cozzone AJ , Duclos B . Phosphorylation of the virulence regulator SarA modulates its ability to bind DNA in *Staphylococcus aureus* . FEMS Microbiol Lett. 2010;306:30–36. 20337713 10.1111/j.1574-6968.2010.01930.x

[29] Sun F , Ding Y , Ji Q , Liang Z , Deng X , Wong CCL , et al. Protein cysteine phosphorylation of SarA/MgrA family transcriptional regulators mediates bacterial virulence and antibiotic resistance. Proc Natl Acad Sci USA. 2012;109:15461–15466. 22927394 10.1073/pnas.1205952109 PMC3458358

[30] Wang G , Li L , Wang X , Li X , Zhang Y , Yu J , et al. Hypericin enhances β‐lactam antibiotics activity by inhibiting *sarA* expression in methicillin‐resistant *Staphylococcus aureus* . Acta Pharm Sin B. 2019;9:1174–1182. 31867163 10.1016/j.apsb.2019.05.002 PMC6900551

[31] Zhong Z , Zhou S , Liang Y , Wei Y , Li Y , Long T , et al. Natural flavonoids disrupt bacterial iron homeostasis to potentiate colistin efficacy. Sci Adv. 2023;9:eadg4205. 37294761 10.1126/sciadv.adg4205 PMC10256158

[32] McCarthy H , Rudkin JK , Black NS , Gallagher L , O'Neill E , O'Gara JP . Methicillin resistance and the biofilm phenotype in *Staphylococcus aureus* . Front Cell Infect Microbiol. 2015;5:1. 25674541 10.3389/fcimb.2015.00001 PMC4309206

[33] Matsuura M , Nakazawa H , Hashimoto T , Mitsuhashi S . Combined antibacterial activity of amoxicillin with clavulanic acid against ampicillin‐resistant strains. Antimicrob Agents Chemother. 1980;17:908–911. 6967713 10.1128/aac.17.6.908 PMC283901

[34] Liu Y , Tong Z , Shi J , Li R , Upton M , Wang Z . Drug repurposing for next‐generation combination therapies against multidrug‐resistant bacteria. Theranostics. 2021;11:4910–4928. 33754035 10.7150/thno.56205 PMC7978324

[35] Liu S , Zhang J , Zhou Y , Hu N , Li J , Wang Y , et al. Pterostilbene restores carbapenem susceptibility in New Delhi metallo‐β‐lactamase‐producing isolates by inhibiting the activity of New Delhi metallo‐β‐lactamases. Br J Pharmacol. 2019;176:4548–4557. 31376166 10.1111/bph.14818 PMC6932935

[36] Liu Y , Jia Y , Yang K , Li R , Xiao X , Zhu K , et al. Metformin restores tetracyclines susceptibility against multidrug‐resistant bacteria. Adv Sci. 2020;7:1902227. 10.1002/advs.201902227 PMC7312304 32596101

[37] Di Pasqua R , Betts G , Hoskins N , Edwards M , Ercolini D , Mauriello G . Membrane toxicity of antimicrobial compounds from essential oils. J Agricult Food Chem. 2007;55:4863–4870. 10.1021/jf0636465 17497876

[38] Archer NK , Mazaitis MJ , Costerton JW , Leid JG , Powers ME , Shirtliff ME . *Staphylococcus aureus* biofilms: properties, regulation, and roles in human disease. Virulence. 2011;2:445–459. 21921685 10.4161/viru.2.5.17724 PMC3322633

[39] Boles BR , Thoendel M , Roth AJ , Horswill AR . Identification of genes involved in polysaccharide‐independent *Staphylococcus aureus* biofilm formation. PLoS One. 2010;5:e10146. 20418950 10.1371/journal.pone.0010146 PMC2854687

[40] Snowden JN , Beaver M , Beenken K , Smeltzer M , Horswill AR , Kielian T . *Staphylococcus aureus sarA* regulates inflammation and colonization during central nervous system biofilm formation. PLoS One. 2013;8:e84089. 24386336 10.1371/journal.pone.0084089 PMC3875531

[41] Beenken KE , Blevins JS , Smeltzer MS . Mutation of *sarA* in *Staphylococcus aureus* limits biofilm formation. Infect Immun. 2003;71:4206–4211. 12819120 10.1128/IAI.71.7.4206-4211.2003 PMC161964

[42] O'Neill E , Pozzi C , Houston P , Smyth D , Humphreys H , Robinson DA , et al. Association between methicillin susceptibility and biofilm regulation in *Staphylococcus aureus* isolates from device‐related infections. J Clin Microbiol. 2007;45:1379–1388. 17329452 10.1128/JCM.02280-06 PMC1865887

[43] Trotonda P , Manna AC , Cheung AL , Lasa I , Penadés R . SarA positively controls Bap‐dependent biofilm formation in *Staphylococcus aureus* . J Bacteriol. 2005;187:5790–5798. 16077127 10.1128/JB.187.16.5790-5798.2005 PMC1196089

[44] Conlon BP , Rowe SE , Gandt AB , Nuxoll AS , Donegan NP , Zalis EA , et al. Persister formation in *Staphylococcus aureus* is associated with ATP depletion. Nat Microbiol. 2016;1:16051. 10.1038/nmicrobiol.2016.51 PMC4932909 27572649

[45] Seidl K , Solis NV , Bayer AS , Hady WA , Ellison S , Klashman MC , et al. Divergent responses of different endothelial cell types to infection with *Candida albicans* and *Staphylococcus aureus* . PLoS One. 2012;7:e39633. 22745797 10.1371/journal.pone.0039633 PMC3382135

[46] Xiong YQ , Bayer AS , Yeaman MR , Van Wamel W , Manna AC , Cheung AL . Impacts of *sarA* and *agr* in *Staphylococcus aureus* strain Newman on fibronectin‐binding protein A gene expression and fibronectin adherence capacity in vitro and in experimental infective endocarditis. Infect Immun. 2004;72:1832–1836. 14977998 10.1128/IAI.72.3.1832-1836.2004 PMC356038

[47] Wolz C , Pöhlmann‐Dietze P , Steinhuber A , Chien YT , Manna A , van Wamel W , et al. *Agr*‐independent regulation of fibronectin‐binding protein(s) by the regulatory locus *sar* in *Staphylococcus aureus* . Mol Microbiol. 2000;36:230–243. 10760180 10.1046/j.1365-2958.2000.01853.x

[48] Mrak LN , Zielinska AK , Beenken KE , Mrak IN , Atwood DN , Griffin LM , et al. *saeRS* and *sarA* act synergistically to repress protease production and promote biofilm formation in *Staphylococcus aureus* . PLoS One. 2012;7:e38453. 22685571 10.1371/journal.pone.0038453 PMC3369899

[49] Li L , Wang G , Cheung A , Abdelhady W , Seidl K , Xiong YQ . MgrA governs adherence, host cell interaction, and virulence in a murine model of bacteremia due to *Staphylococcus aureus* . J Infect Dis. 2019;220:1019–1028. 31177268 10.1093/infdis/jiz219 PMC6688059

[50] Lei MG , Gudeta DD , Luong TT , Lee CY . MgrA negatively impacts *Staphylococcus aureus* invasion by regulating capsule and FnbA. Infect Immun. 2019;87:e0059019. 10.1128/IAI.00590-19 PMC6867852 31591167

[51] Rossiter SE , Fletcher MH , Wuest WM . Natural products as platforms to overcome antibiotic resistance. Chem Rev. 2017;117:12415–12474. 28953368 10.1021/acs.chemrev.7b00283 PMC5869711

[52] Lister PD , Sanders CC . Comparison of ampicillin‐sulbactam regimens simulating 1.5‐ and 3.0‐gram doses to humans in treatment of *Escherichia coli* bacteremia in mice. Antimicrob Agents Chemother. 1995;39:930–936. 7785998 10.1128/aac.39.4.930 PMC162656

[53] English AR , Girard D , Haskell SL . Pharmacokinetics of sultamicillin in mice, rats, and dogs. Antimicrob Agents Chemother. 1984;25:599–602. 6329091 10.1128/aac.25.5.599 PMC185595

[54] Sauve C , Azoulay‐Dupuis E , Moine P , Darras‐Joly C , Rieux V , Carbon C , et al. Efficacies of cefotaxime and ceftriaxone in a mouse model of pneumonia induced by two penicillin‐ and cephalosporin‐resistant strains of *Streptococcus pneumoniae* . Antimicrob Agents Chemother. 1996;40:2829–2834. 9124850 10.1128/aac.40.12.2829 PMC163631

[55] Spentzas T , Kudumula R , Acuna C , Talati AJ , Ingram KC , Savorgnan F , et al. Role of bacterial components in macrophage activation by the LAC and MW2 strains of community‐associated, methicillin‐resistant *Staphylococcus aureus* . Cell Immunol. 2011;269:46–53. 21458780 10.1016/j.cellimm.2011.03.009

[56] Memmi G , Filipe SR , Pinho MG , Fu Z , Cheung A . *Staphylococcus aureus* PBP4 is essential for β‐lactam resistance in community‐acquired methicillin‐resistant strains. Antimicrob Agents Chemother. 2008;52:3955–3966. 18725435 10.1128/AAC.00049-08 PMC2573147

[57] CLSI . Performance standards for antimicrobial susceptibility testing. 31st ed. Wayne, PA: CLSI supplement M100. Clinical and Laboratory Standards Institute; 2021. 10.1128/JCM.00213-21 PMC8601225 34550809

[58] Zhou YF , Xiong YQ , Tao MT , Li L , Bu MX , Sun J , et al. Increased activity of linezolid in combination with rifampicin in a murine pneumonia model due to MRSA. J Antimicrob Chemother. 2018;73:1899–1907. 29897466 10.1093/jac/dky129

[59] Odds FC . Synergy, antagonism, and what the chequerboard puts between them. J Antimicrob Chemother. 2003;52:1. 12805255 10.1093/jac/dkg301

[60] Sedlmayer F , Hell D , Müller M , Ausländer D , Fussenegger M . Designer cells programming quorum‐sensing interference with microbes. Nat Commun. 2018;9:1822. 29739943 10.1038/s41467-018-04223-7 PMC5940823

[61] Poonacha N , Nair S , Desai S , Tuppad D , Hiremath D , Mohan T , et al. Efficient killing of planktonic and biofilm‐embedded coagulase‐negative staphylococci by bactericidal protein P128. Antimicrob Agents Chemother. 2017;61:e0045717. 10.1128/AAC.00457-17 PMC5527639 28559263

[62] Fluckiger U , Ulrich M , Steinhuber A , Döring G , Mack D , Landmann R , et al. Biofilm formation, *icaADBC* transcription, and polysaccharide intercellular adhesin synthesis by staphylococci in a device‐related infection model. Infect Immun. 2005;73:1811–1819. 15731082 10.1128/IAI.73.3.1811-1819.2005 PMC1064907

[63] Chen L , Ren Z , Zhou X , Zeng J , Zou J , Li Y . Inhibition of *Streptococcus mutans* biofilm formation, extracellular polysaccharide production, and virulence by an oxazole derivative. Appl Microbiol Biotechnol. 2016;100:857–867. 26526453 10.1007/s00253-015-7092-1

[64] Rice KC , Mann EE , Endres JL , Weiss EC , Cassat JE , Smeltzer MS , et al. The *cidA* murein hydrolase regulator contributes to DNA release and biofilm development in *Staphylococcus aureus* . Proc Natl Acad Sci USA. 2007;104:8113–8118. 17452642 10.1073/pnas.0610226104 PMC1876580

[65] Banner MA , Cunniffe JG , Macintosh RL , Foster TJ , Rohde H , Mack D , et al. Localized tufts of fibrils on *Staphylococcus epidermidis* NCTC 11047 are comprised of the accumulation‐associated protein. J Bacteriol. 2007;189:2793–2804. 17277069 10.1128/JB.00952-06 PMC1855787

[66] Yuan Z , Dai Y , Ouyang P , Rehman T , Hussain S , Zhang T , et al. Thymol inhibits biofilm formation, eliminates pre‐existing biofilms, and enhances clearance of methicillin‐resistant *Staphylococcus aureus* (MRSA) in a mouse peritoneal implant infection model. Microorganisms. 2020;8:99. 31936809 10.3390/microorganisms8010099 PMC7023310

[67] Yeaman MR , Puentes SM , Norman DC , Bayer AS . Partial characterization and staphylocidal activity of thrombin‐induced platelet microbicidal protein. Infect Immun. 1992;60:1202–1209. 1541535 10.1128/iai.60.3.1202-1209.1992 PMC257613

[68] Shao ES , Lin L , Yao Y , Boström KI . Expression of vascular endothelial growth factor is coordinately regulated by the activin‐like kinase receptors 1 and 5 in endothelial cells. Blood. 2009;114:2197–2206. 19506300 10.1182/blood-2009-01-199166 PMC2744576

[69] Seidl K , Bayer AS , Fowler, Jr. VG , McKinnell JA , Abdel Hady W , Sakoulas G , et al. Combinatorial phenotypic signatures distinguish persistent from resolving methicillin‐resistant *Staphylococcus aureus* bacteremia isolates. Antimicrob Agents Chemother. 2011;55:575–582. 21098242 10.1128/AAC.01028-10 PMC3028773

[70] Selvaraj A , Jayasree T , Valliammai A , Pandian SK . Myrtenol attenuates MRSA biofilm and virulence by suppressing *sarA* expression dynamism. Front Microbiol. 2019;10:2027. 31551964 10.3389/fmicb.2019.02027 PMC6737500

[71] Liu Y , Tong Z , Shi J , Jia Y , Deng T , Wang Z . Reversion of antibiotic resistance in multidrug‐resistant pathogens using non‐antibiotic pharmaceutical benzydamine. Commun Biol. 2021;4:1328. 34824393 10.1038/s42003-021-02854-z PMC8616900

[72] Cui CY , He Q , Jia QL , Li C , Chen C , Wu XT , et al. Evolutionary trajectory of the tet(X) family: critical residue changes towards high‐level tigecycline resistance. mSystems. 2021;6:e0005021. 10.1128/mSystems.00050-21 PMC8269203 34006624

[73] Li L , Wang Q , Zhang H , Yang M , Khan MI , Zhou X . Sensor histidine kinase is a β‐lactam receptor and induces resistance to β‐lactam antibiotics. Proc Natl Acad Sci USA. 2016;113:1648–1653. 26831117 10.1073/pnas.1520300113 PMC4760793

[74] Koo WWK , Ke J , Tam YK , Finegan BA , Marriage B . Pharmacokinetics of ampicillin during parenteral nutrition. J Parenter Enteral Nutr. 1990;14:279–282. 10.1177/0148607190014003279 2112643

[75] Patel KB , Nicolau DP , Nightingale CH , Quintiliani R . Pharmacokinetics of cefotaxime in healthy volunteers and patients. Diagn Microbiol Infect Dis. 1995;22:49–55. 7587050 10.1016/0732-8893(95)00072-i

[76] Bayer AS , Abdelhady W , Li L , Gonzales R , Xiong YQ . Comparative efficacies of tedizolid phosphate, linezolid, and vancomycin in a murine model of subcutaneous catheter‐related biofilm infection due to methicillin‐susceptible and ‐resistant *Staphylococcus aureus* . Antimicrob Agents Chemother. 2016;60:5092–5096. 27297485 10.1128/AAC.00880-16 PMC4958216

[77] Zhou YF , Shi W , Yu Y , Tao MT , Xiong YQ , Sun J , et al. Pharmacokinetic/pharmacodynamic correlation of cefquinome against experimental catheter‐associated biofilm infection due to *Staphylococcus aureus* . Front Microbiol. 2016;6:1513. 26779167 10.3389/fmicb.2015.01513 PMC4703793

[78] Zhou YF , Tao MT , He YZ , Sun J , Liu YH , Liao XP . In vivo bioluminescent monitoring of therapeutic efficacy and pharmacodynamic target assessment of antofloxacin against *Escherichia coli* in a neutropenic murine thigh infection model. Antimicrob Agents Chemother. 2018;62:e0128117. 10.1128/AAC.01281-17 PMC5740385 29038275

[79] Abdelhady W , Bayer AS , Seidl K , Nast CC , Kiedrowski MR , Horswill AR , et al. Reduced vancomycin susceptibility in an in vitro catheter‐related biofilm model correlates with poor therapeutic outcomes in experimental endocarditis due to methicillin‐resistant *Staphylococcus aureus* . Antimicrob Agents Chemother. 2013;57:1447–1454. 23295925 10.1128/AAC.02073-12 PMC3591927

[80] Cheng WX , Zhong S , Meng XB , Zheng NY , Zhang P , Wang Y , et al. Cinnamaldehyde inhibits inflammation of human synoviocyte cells through regulation of Jak/Stat pathway and ameliorates collagen‐induced arthritis in rats. J Pharmacol Exp Ther. 2020;373:302–310. 32029577 10.1124/jpet.119.262907

