# Supplementary material for: Cinnamaldehyde targets SarA to enhance β‐lactam antibiotic activity against methicillin‐resistant Staphylococcus aureus
Source: mLife. 2024 Jun 14;3(2):291–306. doi: 10.1002/mlf2.12121 (PMC11211666; doi:10.1002/mlf2.12121)
Supplement: Supplementary file 1 — Supporting information. [file MLF2-3-291-s002.pdf]

Supplemental Information

Cinnamaldehyde targets SarA to enhance β-lactam antibiotic activity against methicillin-resistant *Staphylococcus aureus*

Jian-Guo Li[1,2], Ting-Yin Lu[1,2], Yue-Fei Chu[1,2], Yue-Jun Zhang[1,2], Jing Zhang[1-3], Wen-Zhen Fu[1,2], Jian Sun[1,2], Ya-Hong Liu[1,2], Xiao-Ping Liao[1,2,#] and Yu-Feng Zhou[1,2,#]

[1] State Key Laboratory for Animal Disease Control and Prevention, South China Agricultural University, Guangzhou, China;

[2] Guangdong Provincial Key Laboratory of Veterinary Pharmaceutics Development and Safety Evaluation, South China Agricultural University, Guangzhou, China;

[3] Yantai Fushan Center for Animal Disease Control and Prevention, Fushan, Yantai, Shandong, China;

[#]**Address correspondence to** Yu-Feng Zhou, zyf@scau.edu.cn and Xiao-Ping Liao, xpliao@scau.edu.cn, College of Veterinary Medicine, South China Agricultural University, Guangzhou, China.

The supplemental information includes:

Table S1. MICs of ampicillin (AMP), cefotaxime (CTX) and cinnamaldehyde (CIN) against MRSA strains and their relevant characteristics.

Table S2. MICs and fractional inhibitory concentration (FIC) indices of AMP, CTX and CIN for clinical MRSA isolates.

Table S3. The differential expression genes (DEGs) in MRSA JE2 treated with cinnamaldehyde (CIN) at 156.25 mg/l (provided in supplemental Excel document).

Figure S1. Time-kill curves depicting *in vitro* potentiation of β-lactam antibiotics by cinnamaldehyde (CIN) for clinical MRSA strains SA-70 and SA-372.

Figure S2. Verification of comparative transcriptome analysis by quantitative real-time PCR (qRT-PCR).

Figure S3. Relative expression levels of *sarA* in MRSA strain JE2 after exposure to cinnamaldehyde (CIN) from 78.12 to 312.5 mg/l.

Figure S4. *In vitro* phosphorylation levels of SarA protein in the absence and presence of cinnamaldehyde (CIN, 156.25 mg/l) alone or in combination with ampicillin (AMP, 8 mg/l) and cefotaxime (CTX, 16 mg/l).

Figure S5. Effects of cinnamaldehyde (CIN, 1.56-12.5 g/l) on the safety of ampicillin (AMP) and cefotaxime (CTX).

Figure S6. Histological evaluations of *in vivo* nephrotoxicity of cinnamaldehyde (CIN, 20 mg/kg) or cefotaxime (CTX, 100 mg/kg) administered alone or in combination by haematoxylin and eosin (H&E) staining.

Figure S7. Densities of MRSA strain MW2 in kidney, spleen and blood after a 3-day β-lactam antibiotic monotherapy or in combination with cinnamaldehyde (CIN) in the murine bacteremia model**.**

Table S1. MICs of ampicillin (AMP), cefotaxime (CTX) and cinnamaldehyde (CIN) against MRSA strains and their relevant characteristics.

MRSA strains	MIC (mg/l)			Relevant genotypes and characteristics	Source or references
	AMP	CTX	CIN		
JE2	16	32	312.5	Plasmid-cured derivative of LAC strain, CA-MRSA (SCC*mec* type IV), USA300	[1]
JE2 Δ*mecA*	0.25	2	312.5	Transposon mutant with insertion in *S. aureus* USA300_0032	NTML[2]
JE2 Δ*sarA*	4	8	312.5	JE2 *sarA::kan* with ALC2543 (COL with a Δ*sarA::kan* mutation)	[3, 4]
JE2 Δ*sarA*/p*sarA*	8	32	312.5	JE2 Δ*sarA* complemented with the plasmid pALC1215 (pSPT181 carrying the entire *sarA* locus)	[2]
JE2 Δ*sarA*/p*mecA*	8	32	312.5	JE2 Δ*sarA* complemented with the plasmid pALC6185 (pEPSA5 containing the *mecA* coding region)	[2, 5, 6]
MW2	8	16	312.5	CA-MRSA (SCCmec type IVa), USA400	[1, 4]

Abbreviations: MIC, minimal inhibitory concentration; AMP, ampicillin; CTX, cefotaxime; CIN, cinnamaldehyde; CA-MRSA, community-acquired methicillin-resistant *S. aureus*; NTML, Nebraska Transposon Mutant Library.

Table S2. MICs and fractional inhibitory concentration (FIC) indices of AMP, CTX and CIN for clinical MRSA isolates.

Strains	MIC (mg/l)			FIC index		Background
	CIN	AMP	CTX	AMP + CIN	CTX + CIN	
SA-70	625	128	32	0.25	0.5	wound pus
SA-71	625	256	32	0.5	0.5	wound pus
SA-72	625	4	16	0.3125	0.375	wound pus
SA-75	625	128	32	0.28125	0.5	wound pus
SA-88	625	16	128	0.3125	0.375	wound pus
SA-264	625	256	32	0.5	0.26525	wound pus
SA-272	625	256	64	0.3125	0.375	wound pus
SA-273	1250	32	512	0.5	0.375	endotracheal aspirate
SA-372	312.5	512	32	0.5	0.3125	human bacteremia
SA-373	625	32	256	0.5	0.5	human bacteremia
SA-379	625	32	256	0.375	0.5	endotracheal aspirate
SA-520	625	16	64	0.5	0.375	human bacteremia
SA-526	625	256	64	0.3125	0.28125	endotracheal aspirate
SA-527	625	32	512	0.28125	0.28125	endotracheal aspirate
SA-595	625	256	32	0.26525	0.3125	human bacteremia
161402	625	32	512	0.5	0.375	endotracheal aspirate; *cfr*-positive (LZD MIC=8 mg/l)[7]
161494	625	32	256	0.5	0.28125	endotracheal aspirate; *cfr*-positive (LZD MIC=4 mg/l)[7]
161429	625	32	256	0.5	0.265625	endotracheal aspirate; *cfr*-positive (LZD MIC=8 mg/l)[7]
MU50	625	0.25	2	0.3125	0.5	ATCC 700699; VISA isolate
43300	625	64	16	0.26525	0.28125	ATCC 43300; MRSA isolate

Abbreviations: AMP, ampicillin; CTX, cefotaxime; CIN, cinnamaldehyde; LZD, linezolid; VISA, vancomycin-intermediate *S. aureus*.

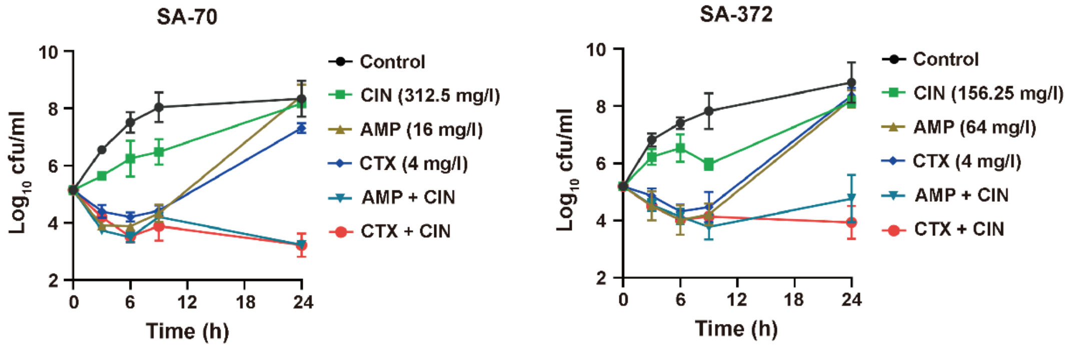

Figure S1. Time-kill curves depicting *in vitro* potentiation of β-lactam antibiotics by cinnamaldehyde (CIN) for clinical MRSA strains SA-70 and SA-372. Exponentially growing MRSA cells were challenged with sub-MIC concentrations of CIN alone and in combination with ampicillin (AMP) or cefotaxime (CTX). Data represent values from three biological replicates.

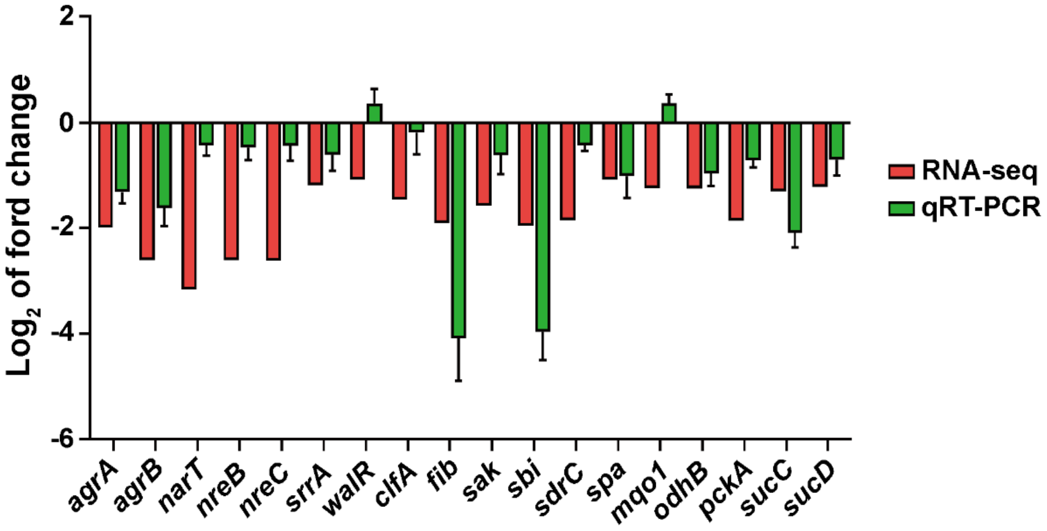

Figure S2. Verification of comparative transcriptome analysis by quantitative real-time PCR (qRT-PCR).

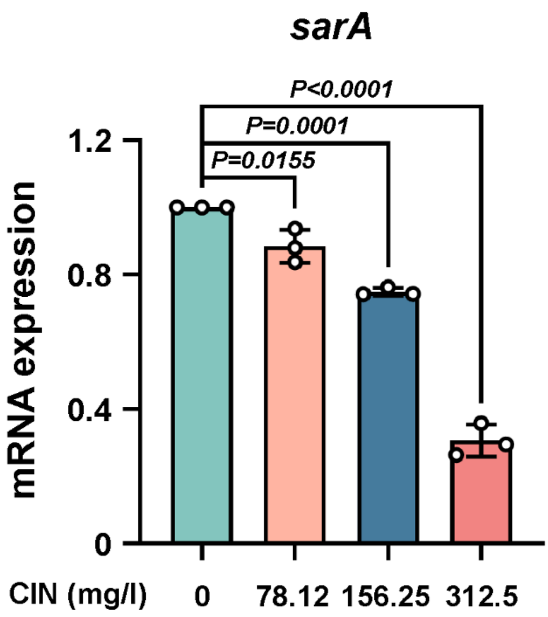

Figure S3. Relative expression levels of *sarA* in MRSA strain JE2 after exposure to cinnamaldehyde (CIN) from 78.12 to 312.5 mg/l. Statistical comparisons were tested by unpaired Student's t test. $P \leq 0.5$ was considered as statistically significant.

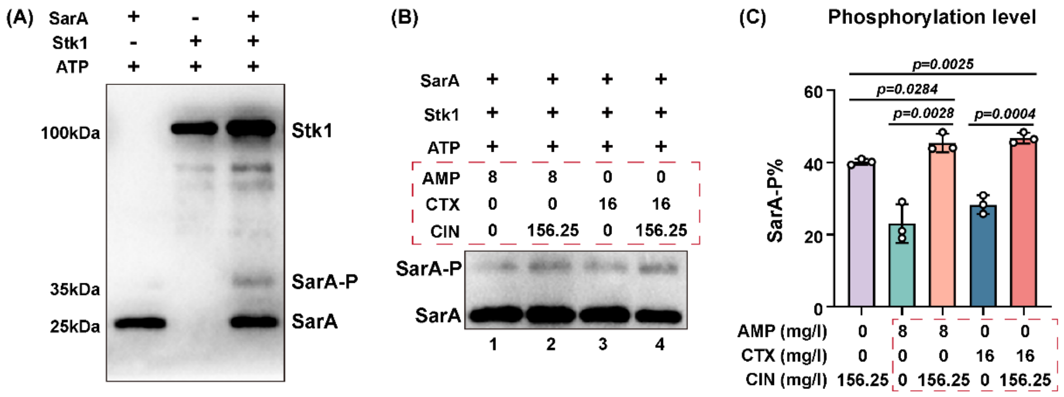

Figure S4. *In vitro* phosphorylation levels of SarA protein in the absence (**A**) and presence (**B, C**) of cinnamaldehyde (CIN, 156.25 mg/l) alone or in combination with ampicillin (AMP, 8 mg/l) and cefotaxime (CTX, 16 mg/l). Statistical comparisons were tested by the unpaired Student's t test, and $P \leq 0.5$ was considered as statistically significant.

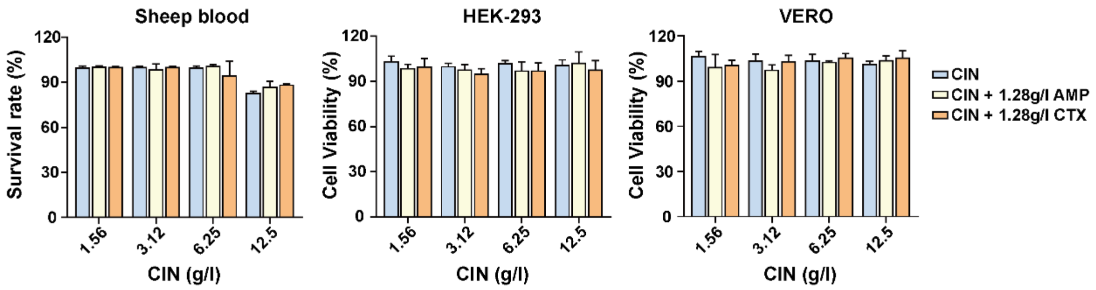

Figure S5. Effects of cinnamaldehyde (CIN, 1.56-12.5 g/l) on the safety of ampicillin (AMP) and cefotaxime (CTX). Safety assessments for CIN on sheep red blood cells, HEK-293 and Vero cells showed negligible hemolytic and cytotoxic activities. Data are represented as mean ± SD of at least three biological replicates.

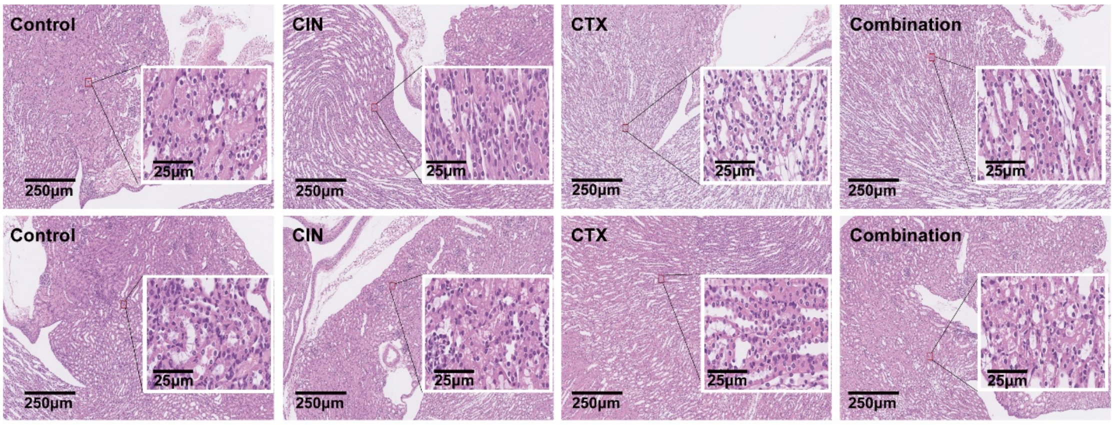

Figure S6. Histological evaluations of *in vivo* nephrotoxicity of cinnamaldehyde (CIN, 20 mg/kg) or cefotaxime (CTX, 100 mg/kg) administered alone or in combination by haematoxylin and eosin (H&E) staining. Panels above and below are representative of two independent replicates.

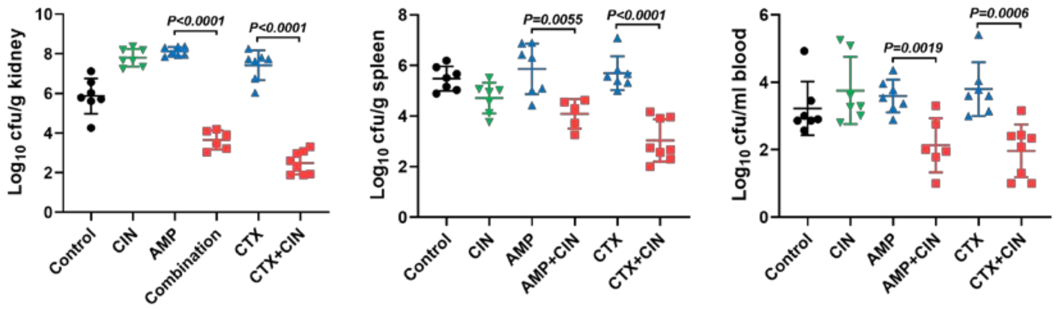

Figure S7. Densities of MRSA strain MW2 in kidney, spleen and blood after a 3-day β-lactam antibiotic monotherapy or in combination with CIN in the murine bacteremia model (CIN at 20 mg/kg, i.v., qd; AMP at 40 mg/kg, p.o., tid; CTX at 100 mg/kg, s.c., bid). Each dots represent one mouse, and horizontal lines indicate the mean of results from groups of six to eight mice. Statistical comparisons were tested by the unpaired Student's t test, and $P \leq 0.5$ was considered as statistically significant. Abbreviations: CIN, cinnamaldehyde; AMP, ampicillin, CTX, cefotaxime; i.v., intravenously; p.o., orally; s.c., subcutaneously; qd, once daily; bid, twice daily; tid, three times daily.

References

1. Spentzas T, Kudumula R, Acuna C, Talati AJ, Ingram KC, Savorgnan F, et al. Role of bacterial components in macrophage activation by the LAC and MW2 strains of community-associated, methicillin-resistant *Staphylococcus aureus*. *Cell Immunol*. 2011; 269:46-53.
2. Li L, Cheung A, Bayer AS, Chen L, Abdelhady W, Kreiswirth BN, et al. The global regulon *sarA* regulates beta-lactam antibiotic resistance in methicillin-resistant *Staphylococcus aureus in vitro* and in endovascular infections. *J Infect Dis*. 2016; 214:1421-9.
3. Abdelhady W, Bayer AS, Seidl K, Moormeier DE, Bayles KW, Cheung A, et al. Impact of vancomycin on *sarA*-mediated biofilm formation: role in persistent endovascular infections due to methicillin-resistant *Staphylococcus aureus*. *J Infect Dis*. 2014; 209:1231-40.
4. Trotonda MP, Xiong YQ, Memmi G, Bayer AS, Cheung AL. Role of *mgrA* and *sarA* in methicillin-resistant *Staphylococcus aureus* autolysis and resistance to cell wall-active antibiotics. *J Infect Dis*. 2009; 199:209-18.
5. Memmi G, Filipe SR, Pinho MG, Fu Z, Cheung A. *Staphylococcus aureus* PBP4 is essential for beta-lactam resistance in community-acquired methicillin-resistant strains. *Antimicrob Agents Chemother*. 2008; 52:3955-66.
6. Wang G, Li L, Wang X, Li X, Zhang Y, Yu J, et al. Hypericin enhances beta-lactam antibiotics activity by inhibiting *sarA* expression in methicillin-resistant *Staphylococcus aureus*. *Acta Pharm Sin B*. 2019; 9:1174-82.
7. Zhou YF, Xiong YQ, Tao MT, Li L, Bu MX, Sun J, et al. Increased activity of linezolid in combination with rifampicin in a murine pneumonia model due to MRSA. *J Antimicrob Chemother*. 2018; 73:1899-907.
